# Autonomous digital lifestyle intervention for obesity: a comparative study of software-generated vs. provider-delivered body composition outcomes

**DOI:** 10.3389/fdgth.2026.1874279

**Published:** 2026-10-23

**Authors:** Ioanna Pagani, Maria Syed, Cynthia Ages, Michael Bell, Yannis Raftopoulos

**Affiliations:** 1 Department of Surgery, Holyoke Medical Center, Holyoke, MA, United States; 2 Department of Surgery, Boundless Apps LLC, Los Angeles, CA, United States

**Keywords:** body composition (BC), body mass index (BMI), daily protein intake goal (DPIG), digital health, fat mass loss (FML), generalized estimated equations (GEE) models, lifestyle intervention (LI), metabolic and bariatric surgery (MBS)

## Abstract

**Background:**

Many lifestyle intervention apps for weight management rely on human coaching, which increases cost and variability, or use calorie-counting approaches that lack structured, individualized guidance. Fully autonomous digital platforms that deliver personalized nutrition and exercise plans with body composition tracking remain limited.

**Objective:**

To compare an autonomous, protein-focused digital lifestyle intervention (RightBMI App) with provider-delivered lifestyle intervention for weight loss and body composition outcomes in metabolic and bariatric surgery (MBS) candidates.

**Materials and methods:**

This prospective quasi-experimental study enrolled 160 patients at a single center (April 2024–April 2025). Patients were sequentially allocated to the RightBMI App (*n* = 80) or provider-delivered care (*n* = 80). Outcomes (% total body weight loss [%TBWL], % fat mass loss [%FML], % visceral fat loss [%VFL], and % muscle mass loss [%MML]) were assessed over 24 weeks using Generalized Estimating Equations (GEE) with multiple imputation and inverse probability weighting, adjusting for age, Body Mass Index (BMI), and sex. A time-to-event analysis (Kaplan–Meier and Cox proportional hazards) was performed as the primary sensitivity analysis to address informative censoring due to MBS clearance at ≥10% TBWL.

**Results:**

In GEE models, the App group showed significantly greater %TBWL at weeks 12 (+2.64%, *p* < 0.001) and 16 (+2.47%, *p* = 0.027), and greater %FML at weeks 12 (+2.94%, *p* = 0.028) and 16 (+4.20%, *p* = 0.003) compared with provider-delivered care. By week 24, outcomes were comparable (%TBWL: 20.01% vs. 19.97%; %FML: 36.81% vs. 35.74%). Both groups achieved substantial fat mass loss while preserving muscle mass. Time-to-event analysis showed no significant difference in time to ≥10% TBWL (log-rank *p* = 0.23; adjusted HR 0.83, *p* = 0.43). The App group had a higher MBS clearance rate by week 24 (90% vs. 71.25%, *p* = 0.0023) with low attrition. User satisfaction was high (mean 8.76/10).

**Conclusion:**

The autonomous RightBMI App demonstrated greater mid-study weight and fat loss compared with provider-delivered care after statistical adjustment, with comparable 24-week outcomes and effective muscle preservation. Time-to-event analysis showed no significant difference in time to achieve ≥10% TBWL. These findings support the potential of fully autonomous digital platforms as scalable alternatives to traditional provider-led interventions in MBS candidates. Limitations include the non-randomized design and use of consumer-grade bioimpedance scales.

## Introduction

Software-based interventions, especially those using machine learning, are proving to be effective tools for helping patients with chronic diseases (1). However, their full potential has yet to be realized (2). While lifestyle intervention (LI) apps can lead to weight loss, their reliance on human coaches or providers has drawbacks, such as increased cost of use, feedback variability, and limited accessibility to underserved populations.

Recent systematic reviews and meta-analyses (2022–2025) confirm that although many contemporary mHealth applications incorporate real-time telemetry, algorithmic feedback, and self-monitoring features, most still rely on human coaching or function primarily as tools for self-monitoring and education rather than as fully autonomous, structured interventions (3–6). Most existing apps are based on calorie-counting guidelines and lack structured, individualized guidance, leaving it to each patient to determine how to adopt and incorporate the provided information into their daily meal and exercise plan. Furthermore, fully autonomous apps that deliver integrated nutrition and exercise planning together with body composition monitoring remain limited (7–10).

### Our solution and findings

To address these limitations, we created an autonomous, protein-focused digital platform that aims to preserve muscle mass while promoting fat loss. It monitors changes in both fat and muscle and provides personalized, structured nutrition and exercise plans. A proof-of-concept study with 275 patients showed that over a 14-week period, those who used our software achieved a statistically significantly greater total body weight loss (TBWL) of 7.61%, compared to 6.07% for patients who received their nutritional plan from a physician experienced in weight management [(11), Conference abstract]. A follow-up study focusing on body composition changes, including 178 patients with a 16-week follow-up, confirmed comparable %TBWL between patients who used the app and those who received a meal and exercise plan by an experienced weight management physician, and demonstrated that app users achieved a %fat mass loss (22.9%) that was twice their %TBWL (11.5%) with no muscle mass loss [(12), Conference abstract]. Furthermore, patients who used the app achieved better %TBWL (7.4% vs. 5.6%) and were able to increase their muscle mass (6% gain vs. 15% loss) compared with patients treated with GLP-1/GIP receptor agonists when both modalities were used as stand-alone therapies [(13), Conference abstract].

In Table 1 we provide a structured comparison of recent digital weight management interventions. Based on this review, RightBMI differs from most existing apps in several important ways: it operates as a fully autonomous platform that does not require ongoing human coaching or provider oversight and delivers structured, dynamically adjusted, individualized meal and exercise plans within a single application, with a strong emphasis on protein intake and muscle preservation. While some recent programs offer personalized plans, few combine full autonomy with integrated body composition tracking focused on substantial fat mass loss while preserving muscle mass. In this study, RightBMI achieved clinically meaningful outcomes, including 19% total body weight loss and 35.8% fat mass loss at 24 weeks with negligible muscle loss. We believe these features support the novelty and potential clinical utility of the RightBMI approach.

**Table 1:** Summary of features and weight loss outcomes from recent reviews on digital interventions for obesity/weight management compared to the RightBMI app.

App/Study	Human interaction/Coaching	Demographics (Ethnicity/Race/SES)	Study design	Control group	Assesses body composition (Fat/Muscle)	Follow-up length	Nutrition plan	Exercise plan	Mean weight loss	Mean BMI reduction	Mean % fat loss	Key notes
AKTIDIET (7, 8)	Yes (1 in-person session + motivational messages)	Spain; limited diversity data	RCT	Minimal intervention	No	6 months	Structured	Structured	∼1–2 kg	0.76	N/A	
SoSu-life (7, 8)	Minimal to none (self-guided with feedback)	Denmark; limited diversity data	RCT	No intervention	Yes (body fat %)	38 weeks	Individualized	Individualized	∼1–2 kg	N/A	0.46–0.79%	Primarily tracking + feedback
My Meal Mate (7, 8)	Yes (1 in-person session)	UK; limited diversity data	RCT	Paper diary + in-person	Yes (body fat %)	6 months	Individualized	Individualized	∼2.2 kg	∼0.8	0.46–0.79%	
Noom Weight (14)	Yes (1:1 coaching + group support)	US; 95% Caucasian; higher education	RCT	Standard care	No	4 months	Individualized	Individualized	5.5 kg	1.5	N/A	Strong coaching component
MyFitnessPal (15)	No (self-guided tracking)	US; mixed race/ethnicity; moderate SES	RCT	Usual care	No	6 months	Calorie tracking (recent planner feature)	Activity tracking	1.8 kg	0.6	N/A	Primarily self-monitoring tool
zanadio (6)	Minimal to moderate (some coaching)	Germany; limited diversity; higher SES bias	RCT	Usual care/minimal	Limited (mainly weight/BMI)	3–12 months	Structured & personalized	Structured	∼3–6 kg	∼1–2	N/A	Closest recent competitor
Oviva Direkt (16)	Yes (professional oversight)	Germany; limited diversity data	Real-world + RCT	Standard care	Yes (some body comp)	Up to 12 months	Personalized	Yes	∼5–8% TBWL	∼1.5–2.5	Limited data	Includes dietitian support
RightBMI App (This study)	**No**—fully autonomous, no provider involvement required	US (Holyoke, MA); Mean age 41 (SD 12); BMI 43 (SD 7.9); 87.5% female; English/Spanish versions; addresses underserved populations	Prospective quasi-experimental with sequential allocation	Provider-delivered lifestyle intervention	**Yes** (fat mass, visceral fat, muscle mass loss)	Up to 24 weeks	**Structured, dynamic, protein-focused individualized plans**	**Structured, individualized**	**24.7 kg -19% TBWL**	**4** **.** **89**	**35.8%**	**Fully autonomous + strong muscle preservation**

*Bold type denotes unique app that provides all 4 features for nutrition: (i) structured, (ii) dynamic, (ii) protein-focused and (iv) individualized meal plans and all 2 features for exercise: (i) structured and (ii) individualized, while being fully autonomous, assessing body composition, and providing strong muscle preservation.

### Study objective

The goal of this study was to compare the weight loss and body composition outcomes between our software-generated and an experienced provider-delivered LI and to evaluate our platform's overall effectiveness.

## Materials and methods

### Study design and group allocation

This was a prospective quasi-experimental study conducted at Holyoke Medical Center between April 2024 and April 2025. Patients evaluated for Metabolic and Bariatric Surgery (MBS) were allocated to one of two groups based on the timing of their enrollment rather than individual randomization. During the first enrollment period, all eligible patients received a lifestyle intervention delivered by a bariatric surgeon or physician assistant (no-App group; *n* = 80). During the subsequent enrollment period, all eligible patients received the lifestyle intervention through the RightBMI mobile application without direct provider involvement (App group; *n* = 80). Because allocation was determined by calendar period rather than random assignment, this was not a randomized controlled trial.

Due to the sequential nature of allocation, the two groups were not expected to be perfectly balanced on all baseline characteristics. The App group was significantly younger and had a higher proportion of male participants than the no-App group. These imbalances were addressed through statistical adjustment in the primary analyses.

The study was not blinded. Patients and the clinical providers delivering care were aware of which intervention they were receiving or delivering. However, researchers responsible for data curation, cleaning, and statistical analysis were not involved in the allocation process and worked with de-identified datasets.

### Eligibility criteria

Patients were eligible for inclusion if they were adults aged >18 years with a body mass index (BMI) > 30 kg/m^2^ who were being evaluated for MBS and expressed interest in participating in a structured lifestyle intervention program, with or without proceeding to surgery. Additional inclusion requirements were a minimum planned participation of 12 weeks in the program, willingness to use one of the study-approved body composition scales for weekly measurements, willingness to incorporate protein shakes and/or protein bars into their nutritional plan, and ownership of a smartphone capable of running the RightBMI application.

Patients were excluded if they had undergone previous MBS, had severe cognitive impairment, had active substance use disorder, had unmanaged major psychiatric disorders, or did not meet any of the inclusion criteria.

### Withdrawal and discontinuation criteria

Participants were withdrawn from the study if they voluntarily left the bariatric program, developed an acute medical condition that precluded continued participation, became pregnant during the study period, or initiated GLP-1/GIP receptor agonist therapy (as this would confound weight loss outcomes).

### RightBMI algorithm description

The RightBMI application operates using a rule-based clinical algorithm developed from over two decades of bariatric surgical and nutritional experience. The algorithm does not employ machine learning or predictive artificial intelligence. Instead, personalization is achieved through structured clinical rules.

Upon registration, patients provide demographic, clinical, and behavioral information, including age, height, weight, sleep/wake times, meal patterns, snacking habits, protein supplement preferences, and physical limitations (Figures 1A–C). The primary function of the algorithm is to calculate an individualized daily protein intake goal of approximately 1 g per kg of ideal body weight (adjusted downward by 10–20 g). This protein target is then dynamically distributed across the patient's reported awake hours and eating patterns to minimize prolonged periods without protein intake.

**Figure 1:**
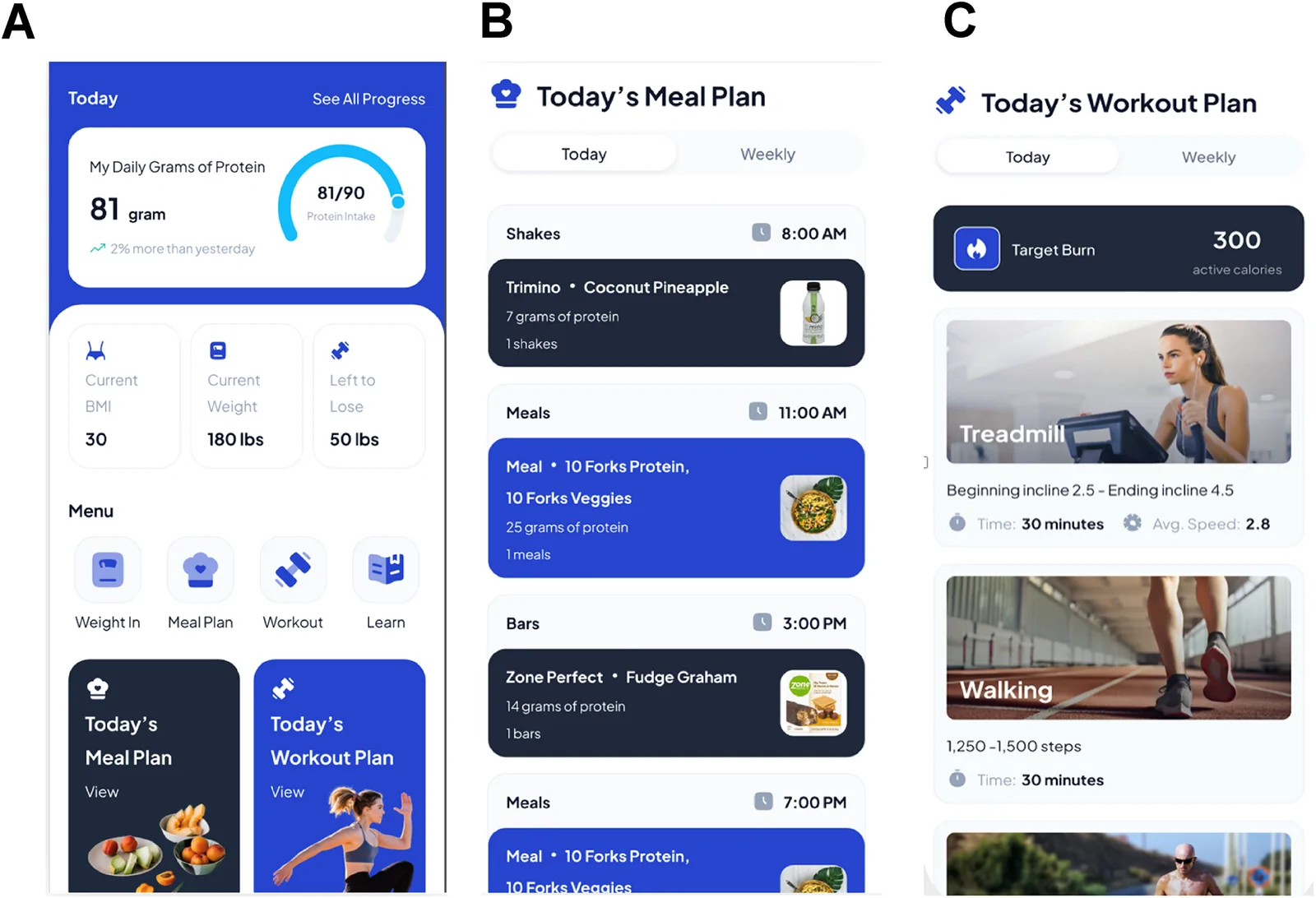
Screencaps of the RightBMI App for **(A)** Main menu **(B)** Meal plan **(C)** Workout plan.

Patients may select from four levels of meal plan “aggressiveness,” ranging from plans using only protein shakes and bars to plans that include up to three food-based meals. The app allocates protein across meals and snacks accordingly (for example, 30% of daily protein to the food meal in 1-food-meal plans and 60%–70% in 2- or 3-food-meal plans). Protein portions are converted into practical visual units (“forkfuls”), where two forkfuls are defined as approximately 30 g of protein containing 7 g of actual protein. Patients also receive equivalent portions of vegetables, with the option to substitute up to half of the vegetable portion with carbohydrates.

The application automatically adjusts the entire plan when patients change protein products, modify their sleep schedule (e.g., night shifts), or lose weight. Exercise recommendations are generated based on age, BMI, and reported musculoskeletal or medical conditions, with appropriate restrictions and reduced intensity goals applied for safety when indicated.

### Rationale for the protein-centric algorithm

The RightBMI algorithm does not isolate protein intake to the exclusion of other nutritional parameters. It is explicitly designed to achieve a controlled overall energy deficit while strategically prioritizing a high protein-to-energy ratio to maximize satiety and preserve lean body mass during rapid preoperative weight loss.

The overall caloric deficit is achieved through a combination of structured protein prioritization, controlled portion sizes, recommended vegetable intake (with optional carbohydrate substitution), multivitamin recommendation, and patient-specific aerobic exercise goals. This multi-component structure aims to support micronutrient adequacy while maintaining the primary therapeutic focus on protein.

This structured, protein-first approach with dynamic, patient-specific implementation was developed from over two decades of bariatric clinical experience and is intended to improve long-term adherence compared with traditional calorie-counting methods. The strategy is supported by evidence that higher protein intake during caloric restriction and in the perioperative bariatric period improves weight loss outcomes, enhances compliance, and reduces loss of fat-free mass (17–19).

### Group protocols

The no-App group received plans generated by a healthcare provider, either a bariatric surgeon or a physician assistant, both with extensive prior experience in weight management. These plans were based on the same principles and algorithms as the RightBMI app, focusing on daily weight-based protein intake distributed throughout the patient's awake time rather than on calorie counting. All patients in the study had not yet undergone MBS at the time of their last follow-up measurement.

To ensure consistent tracking, all patients were asked to use one of four recommended body composition scales and send weekly measurements to their designated provider. Participants who could not afford one of the recommended scales were provided one free of charge by the study. In the App group, patients began by watching three educational video tutorials upon registering. They then provided demographic details, height, weight, and body composition measurements. The app collected medical history and calculated their BMI, personalized weight loss goal, and daily protein intake goal (DPIG). Patients were also asked to report their typical sleep and wake times, meal patterns, snacking habits, protein supplement preferences, and desired pace of weight loss.

Based on this information, the app generated a personalized meal plan with specific mealtimes, meal counts, and portions. Protein and vegetable portions were provided in forkfuls with visual guidance. After providing informed consent and disclosing physical limitations, users proceeded with the exercise planning module, which offered daily workout options with patient-specific aerobic exercise intensity and both daily and weekly calorie burn goals. The exercise module included options that did not require gym equipment or treadmills/ellipticals. The app allowed for flexible, real-time modifications to meal and exercise plans.

### Data collection and transmission

Participants were instructed to perform weekly body composition measurements at home using one of the study-recommended scales. To minimize inter-device variability, a single model (Renpho Bluetooth Smart Scale) was used by 78.2% of participants across both groups, with the remaining participants using other commercial smart scales (Etekcity, GE Fit, iHome, or Runstar). Scale brand distribution did not differ significantly between the App and no-App groups (chi-square test, *p* = 0.78). Each participant maintained use of the same scale model throughout the 24-week study period.

All participants received standardized instructions for at-home measurements through both the application and provider counseling. Patients were instructed to weigh themselves on a consistent weekday morning while fasting, after morning urination, wearing minimal clothing, and with the scale placed on a hard, level surface (hardwood or tile). They were also directed to calibrate the scale monthly and replace batteries regularly to maintain measurement consistency. Although consumer-grade bioimpedance scales have known limitations in absolute precision compared with laboratory methods (20–22), the standardized protocol and consistent device use per participant support the reliability of intra-individual longitudinal trends in body composition.

### Outcome measures

The primary outcome was total body weight loss (%TBWL) from baseline to last recorded follow-up (up to 24 weeks). Secondary outcomes included fat mass loss (%FML), visceral fat loss (%VFL), and muscle mass loss (%MML). All body composition data were measured using one of the recommended bioimpedance scales, and weekly body composition measurements were sent to the providers. For App users, average session duration, return usage rate, bounce rate, and user satisfaction (1–10 scale) were extracted from app usage logs and survey responses.

### Adherence assessment

Patient adherence to the personalized nutrition and exercise plans was evaluated using a combination of application engagement metrics and objective body composition trends derived from weekly scale measurements. Weekly fat mass loss comprising ≥80% of total weekly weight loss was considered indicative of good adherence to the prescribed meal plan. Weekly weight loss > 1.5 lbs (0.68 kg) was interpreted as consistent with adequate exercise plan adherence. A negative change in body water percentage was used as a marker of possible high sodium intake or insufficient exercise intensity. Values falling outside these thresholds triggered automated in-app messages and/or direct communication from the clinical team.

These indicators were reviewed alongside application usage data (session frequency and duration). For patients receiving care within the bariatric practice, adherence was further informed by routine clinical follow-up. This multi-method approach provides practical signals of compliance but does not constitute direct or objective verification of dietary intake or exercise completion. This multi-method approach to assessing adherence in a digital lifestyle intervention is consistent with established frameworks for evaluating digital behavior change interventions (23) and with recent scoping reviews examining how adherence is measured in mHealth physical activity studies (24). The RightBMI platform did not employ food diaries, wearable activity trackers, or biochemical validation to directly confirm protein consumption or exercise completion. Therefore, the body-composition and engagement metrics used in this study should be interpreted as indirect indicators only.

### Power calculation

We performed an *a priori* power calculation for a two-sample comparison of means (two-sided *α* = 0.05) for %TBWL, assuming equal allocation and a common standard deviation of 8% based on the observed variability in the data. Detecting a between-group difference of 5 percentage points with 80% power required approximately 43 participants per group (total *N* = 86). In our study, 61 participants in the App group and 60 participants in the no-App group were available for %TBWL analysis at week 8. With these sample sizes, the study had 91.1% power to detect the prespecified 5 percentage point difference at this time point.

### Statistical methods

%TBWL, %FML, %VFL, and %MML were calculated based on absolute weight loss, fat mass loss, visceral fat loss, and muscle mass loss at each follow-up timepoint in relation to the patient's initial corresponding measurements. Statistical significance thresholds of *p* < 0.05 were set for all calculations, and *p*-values were adjusted with the Benjamini-Hochberg False Discovery Rate method (BH-FDR).

Due to high patient progression rates to MBS after week 8 (as shown in Sankey diagrams, Figures 3A,B), longitudinal comparisons were performed using Generalized Estimating Equations (GEE) with an exchangeable correlation structure, adjusting for age, BMI, and sex. Missing data were addressed through multiple imputation using the MICE algorithm (Multiple Imputation by Chained Equations), and Inverse Probability Weighting (IPW) was applied to account for missingness patterns. A statistical significance threshold of *p* < 0.05 was used, with *p*-values adjusted using the Benjamini-Hochberg False Discovery Rate method.

To assess the influence of age, sex, BMI, and intervention type on outcome variability at week 8, Principal Component Analysis (PCA) was performed (Supplementary Figures 1A–D), with results corroborated using UMAP and t-SNE (results not shown).

Statistical analyses were conducted using Python 3.10.9 with the Pandas, SciPy.stats, Statsmodels, and Fancyimpute libraries. Data visualization was performed using Seaborn and Plotly.

### Time-to-event analysis

Because patients were cleared for MBS upon reaching ≥10% TBWL and subsequently stopped contributing data, creating informative censoring, we performed a time-to-event analysis as the primary sensitivity analysis in the full cohort with baseline data (*n* = 126 in the App group and *n* = 127 in the no-App group), as this approach includes all patients regardless of whether they contributed data at later time points. The event was defined as the first week in which %TBWL reached or exceeded 10%. Patients were followed until the event occurred or were censored at their last available measurement (maximum follow-up 24 weeks). Kaplan–Meier curves were constructed and compared using the log-rank test. Cox proportional hazards regression was used to estimate the hazard ratio for the App vs. no-App group, adjusting for age, BMI, and sex. The proportional hazards assumption was assessed using Schoenfeld residuals; a minor violation was noted for BMI (*p* = 0.048) but was not considered to materially affect interpretation of the primary exposure (App vs. no-App).

### Ethical considerations

This study was approved by an independent Institutional Review Board (WCG) with IRB Tracking Number 20250236. Patient data were de-identified prior to analysis.

## Results

### Participant flow

The flow of participants through the study is shown in Figure 2 (STROBE flow diagram). A total of 324 patients were evaluated for MBS during the study period. Of these, 253 patients were enrolled across the two sequential enrollment periods (127 in the provider-delivered group and 126 in the App group). After excluding patients with no follow-up body composition data, 160 patients (80 per group) were included in the final analysis.

**Figure 2:**
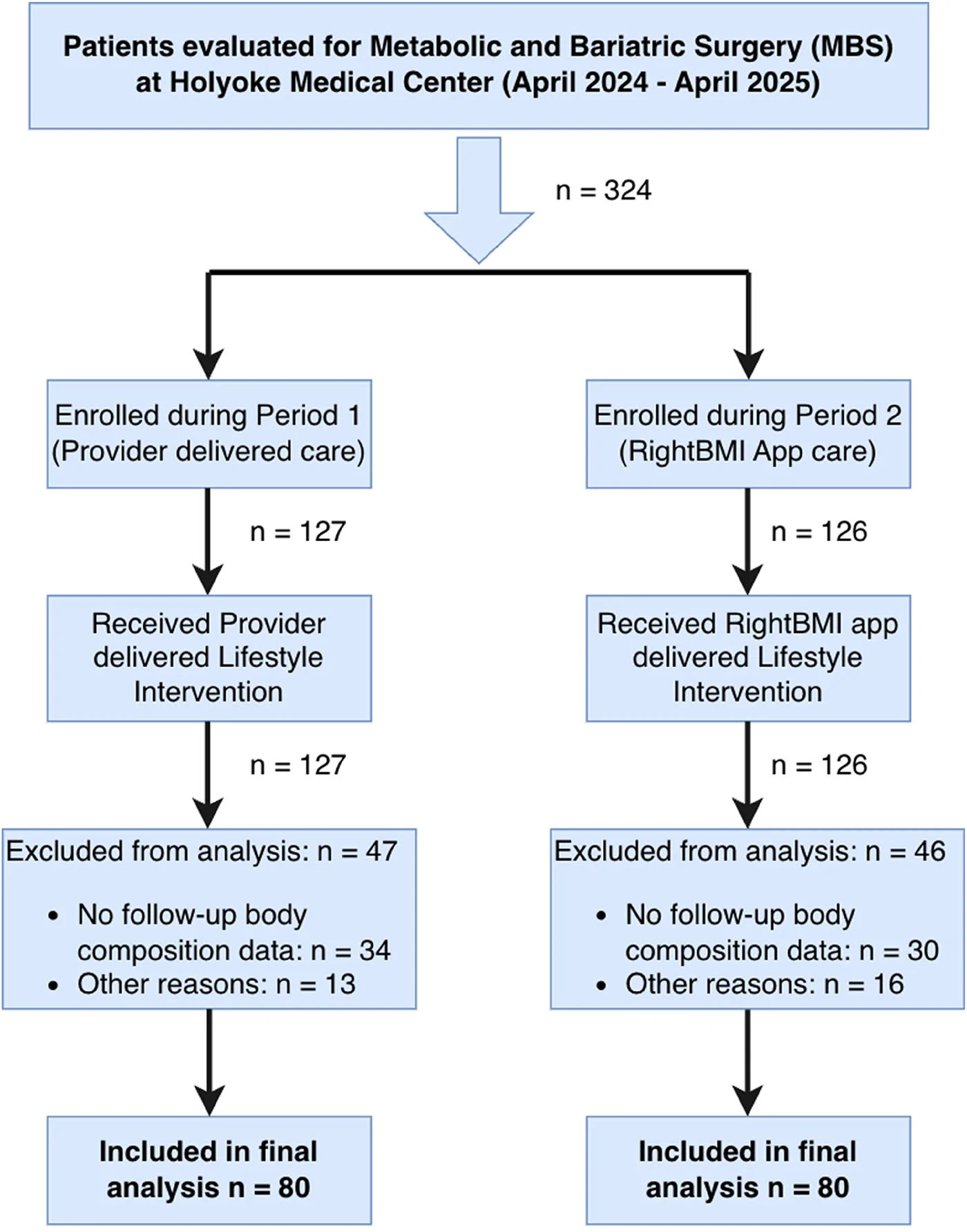
STROBE flow diagram of participant enrollment, allocation, and analysis in a prospective quasi-experimental study comparing our autonomous digital lifestyle intervention RightBMI App with provider-delivered care among patients evaluated for metabolic and bariatric surgery (April 2024–April 2025.

### Baseline characteristics

The App group (*n* = 80) and no-App group (*n* = 80) showed some baseline differences. The App group had a higher proportion of male participants (12.5% vs. 6.25%, *p* < 0.05) and was younger on average (41.09 ± 12.7 years vs. 45.07 ± 11.7 years, *p* < 0.05). Baseline BMI was similar between groups (43.58 ± 7.85 kg/m^2^ vs. 43.05 ± 7.18 kg/m^2^, *p* > 0.05). PCA was used to explore the relative contributions of variables (age, sex, BMI, and intervention type) to variability in outcomes at week 8. Results indicated that intervention type and age were the main factors associated with variance in %TBWL (79.4% and 20.4%, respectively), %FML (78.2% and 21.6%), %VFL (78.0% and 21.9%), and %MML (78.1% and 21.7%). BMI and sex contributed minimally (≤0.1% each) in this analysis (Supplementary Figures 1A–D). These findings were consistent with alternative dimensionality reduction methods (UMAP and t-SNE; results not shown).

### Observed outcomes and progression to MBS

Patient progression to MBS is visualized in Sankey diagrams (Figures 3A,B), showing substantial MBS clearance after week 8 in both groups. By week 20, the App group showed a marked increase in clearance rates (73.75% vs. 66.25% in the provider-led group), which was however not statistically significant (Fisher's exact test *p* = 0.206, odds ratio = 1.43, 95% CI: 0.83-Inf). Notably, by week 24, the App group demonstrated a significantly higher clearance rate (90%) compared to the provider-led group (71.25%) (Fisher's exact test *p* = 0.0023, odds ratio = 3.62, 95% CI: 1.56-Inf). Of the total number of patients in each group only 1.25% dropped out in the no-App group (1 patient at week 8), and 3.75% dropped out in the App group (2 patients at week 8 and 1 at week 12), with Fisher's Exact Test *p* = 0.621, indicating no significant difference in attrition rates between the App and no-App groups. True non-MBS attrition was very low (1.25% in the no-App group and 3.75% in the App group; *p* = 0.621).

**Figure 3:**
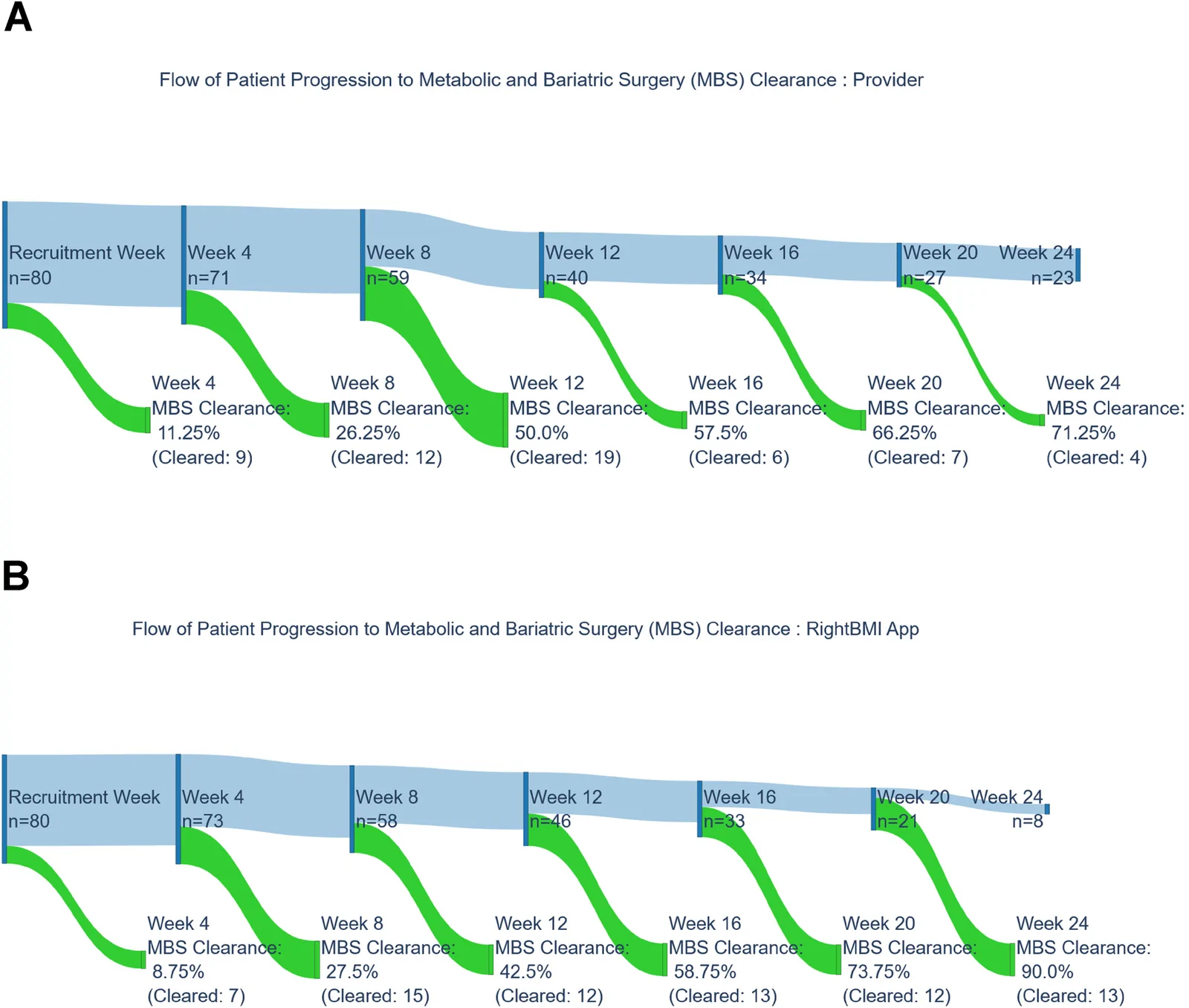
Sankey diagrams of patient progression to MBS from recruitment week to week 24. **(A)** Provider group (no-App) **(B)** RightBMI App group (App). The horizontal layout progresses from Recruitment Week (left) to Week 24 (right). Blue flows represent the number of patients retained between consecutive time points, with node labels indicating the number of patients remaining (e.g., *n* = 80 at Recruitment Week). Green flows indicate the number of patients who were cleared out for MBS between time points, with labels showing the clearance percentage and count [e.g., “Bariatric Surgery Clearance: 50% (Cleared: 19)” for Week 12].

### Unadjusted mean comparisons of outcomes

Unadjusted mean comparisons of outcomes are presented as boxplots in Figures 4A–D (raw, observed cross-sectional data at each time point) and Supplementary Table 1. Sample sizes at each time point are indicated below the *x*-axis, with statistically significant differences between groups marked by asterisks (*p* < 0.05 after Benjamini-Hochberg correction). Mean %TBWL values for the App vs. no-App groups were 3.4% vs. 3.2% at week 4, 6.2% vs. 7.3% at week 8, 8.5% vs. 11.1% at week 12, 11.6% vs. 15.42% at week 16, 15.46% vs. 19.75% at week 20, and 20.01% vs. 19.96% at week 24. However, these Week 24 cross-sectional comparisons are limited by substantial attrition, with only 8 patients remaining in the App group and 22 in the no-App group. In these unadjusted analyses, the no-App group showed significantly higher %TBWL at weeks 12 (*p* = 0.008, BH-corrected *p* = 0.024) and 16 (*p* = 0.005, BH-corrected *p* = 0.024), with a trend at week 20 (*p* = 0.028, BH-corrected *p* = 0.056). No significant differences were observed at weeks 4, 8, or 24.

**Figure 4:**
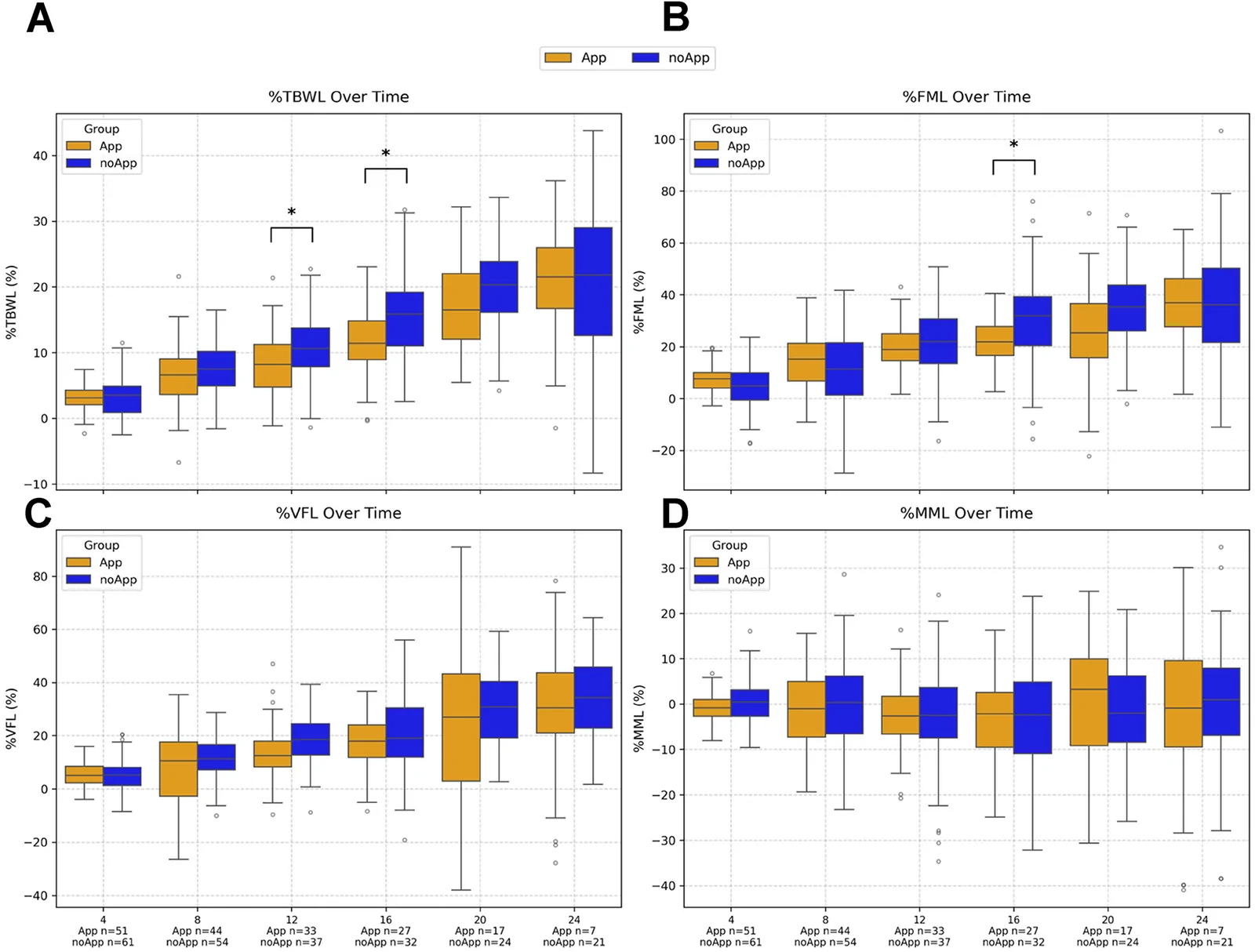
Observed cross-sectional weight loss parameters between weeks 4 and 24 for the App group (orange) vs. the no-App group (blue) for **(A)** %TBWL (%Total Body Weight Loss), **(B)** %FML (%Fat Mass Loss), **(C)** %VFL (%Visceral Fat Loss), **(D)** %MML (%Muscle Mass Loss). Boxplots show raw, observed data (not model-predicted values) at each time point for the App group (orange) versus the no-App group (blue). Sample sizes (n) at each time point are displayed below the *x*-axis. Horizontal brackets with asterisks (*) indicate statistically significant differences between groups at that time point (*p* < 0.05 after Benjamini-Hochberg correction). %TBWL at weeks 12 and 16 was statistically significantly higher for the no-App group but not for any other timepoint. %FML was statistically significantly higher for the no-App group only at week 16, while %MML and %VFL showed no statistically significant difference between the two groups at any time point.

For %FML, the no-App group had significantly higher values at week 16 (28.76% vs. 22.70%, *p* = 0.006, BH-corrected *p* = 0.035), with nonsignificant trends at other time points. At week 24, %FML was similar (App: 36.81%, no-App: 35.74%). %VFL was consistently higher in the no-App group but without statistical significance (e.g., week 24: 31.99% vs. 32.97%, *p* = 0.89). For %MML, the only significant difference was at week 4, where the App group showed lower loss (−1.30% vs. +1.10%, *p* = 0.009, BH-corrected *p* = 0.051); no differences were seen thereafter. Across both groups, %FML and %VFL generally exceeded %TBWL, while %MML remained low and comparable (Figures 4A,B, Supplementary Figure 2).

### Subgroup and correlation analysis

Subgroup analyses within the no-App group (stratified by provider supervision [surgeon vs. PA; Supplementary Figures 3A–D, Supplementary Table 2] and the App group [stratified by operating system (Android vs. iPhone; results not shown)] showed no significant differences in outcomes. At week 8, we performed Spearman correlation analyses to examine relationships between %FML, %VFL, %MML, and %TBWL (Figures 5A,B). The correlation between %FML and %TBWL was strong (Spearman *ρ* = 0.96, *p* = 2.2 × 10^−44^), and %VFL with %TBWL showed a moderate positive correlation (*ρ* = 0.78, *p* = 7.53 × 10^−17^), indicating linear associations. In contrast, %MML showed no significant correlation with %TBWL (*ρ* = −0.07, *p* > 0.05), suggesting that MML does not trend with TBWL.

**Figure 5:**
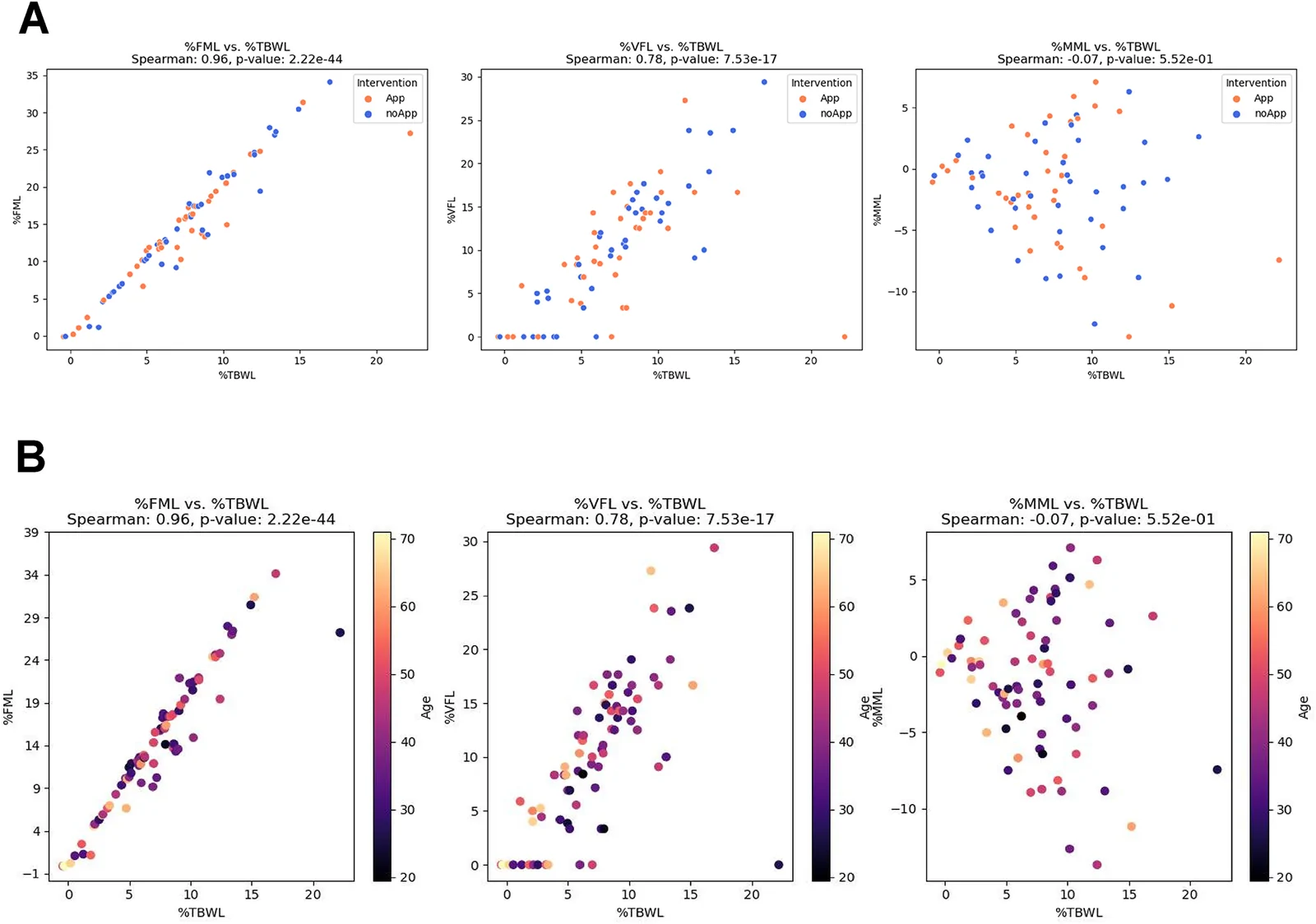
**(A)** Scatter plots of week 8%FML, %VFL and %MML vs. %TBWL for the complete cohort, color-coded for Intervention (App vs. no-App). **(B)** Scatter plots of Week 8%FML, %VFL and %MML vs. %TBWL color-coded for Age. %FML vs. %TBWL Spearman coefficient = 0.96, %VFL vs. %TBWL, Spearman coefficient = 0.78 (*p* < 0.05) while %MML vs. %TBWL Spearman coefficient = −0.07 with *p* > 0.05. Patients from the App group and the no-App group as well as patients from different ages are evenly distributed in the scatter plots, showing no clustering for either different group (App vs. no-App) or age.

### Time-to-event analysis (primary sensitivity analysis)

To address informative censoring due to MBS clearance upon reaching ≥10% TBWL, we conducted a time-to-event analysis in the full cohort with baseline data (*n* = 126 in the App group and *n* = 127 in the no-App group). Thirty-four patients (27.0%) in the App group and 42 patients (33.1%) in the no-App group reached ≥10% TBWL during the observation period. The median time to event was not reached in either group. Kaplan–Meier analysis showed no statistically significant difference in time to ≥10% TBWL between groups (log-rank *p* = 0.2289) (Figure 6). In the adjusted Cox proportional hazards model, the App group had a hazard ratio of 0.83 (*p* = 0.433) compared with the no-App group, indicating no significant difference in the rate of achieving surgical clearance criteria (Supplementary Table 3).

**Figure 6:**
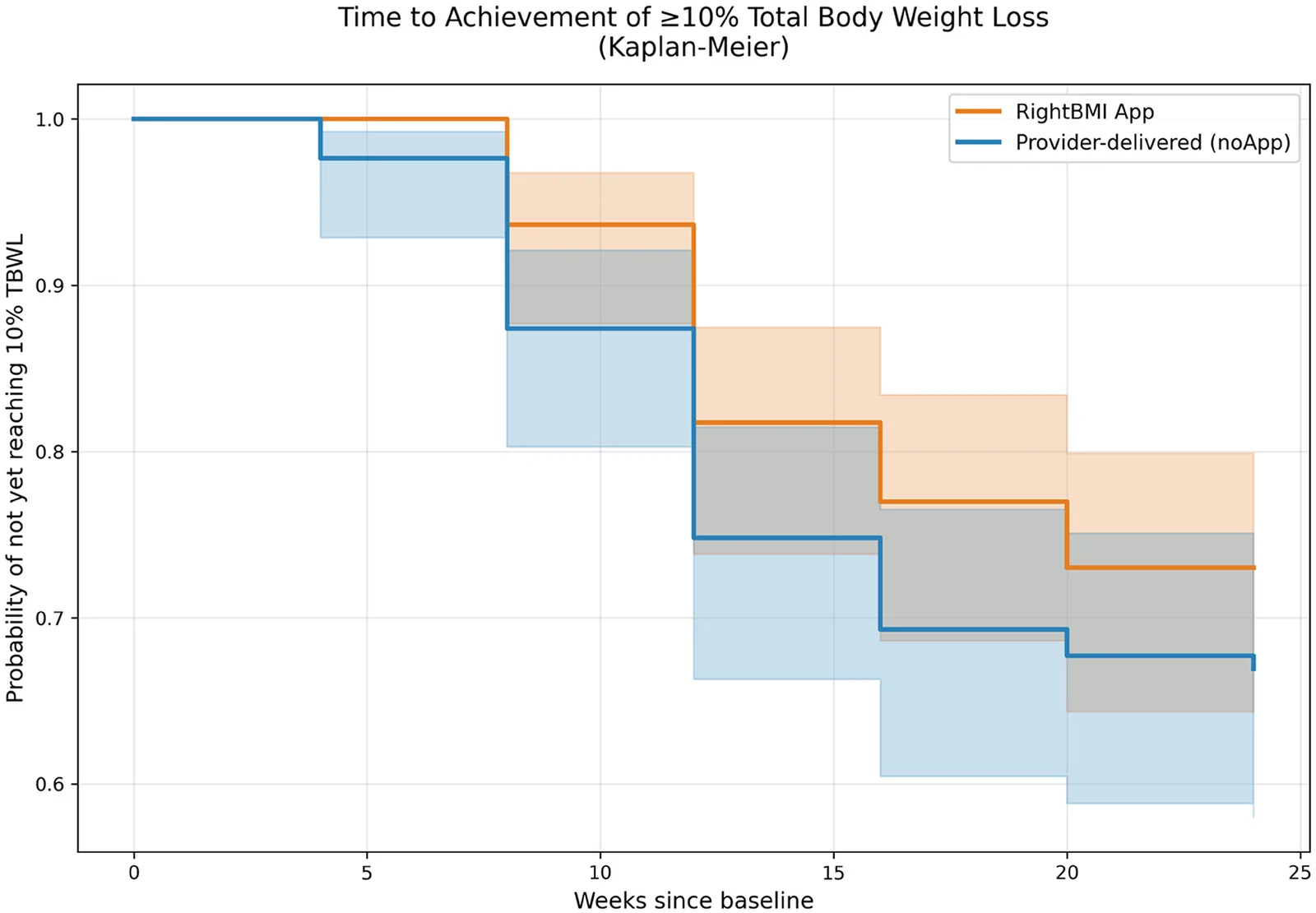
Kaplan–meier curves for time to achievement of ≥10% total body weight loss. Patients in both the App and no-App groups were followed until they reached ≥10% TBWL or were censored at their last available measurement or upon MBS clearance. The log-rank test showed no statistically significant difference between groups (*p* = 0.23).

### Longitudinal GEE models

As unadjusted comparisons do not account for baseline differences (e.g., age and sex) or missing data due to MBS progression, to provide a more robust evaluation, we conducted longitudinal analyses using GEE models with an exchangeable correlation structure, MICE and IPW to handle missing data. Models were adjusted for age, BMI, and sex, allowing inclusion of all available observations rather than complete cases only (Figures 7A–D, Supplementary Table 4). This approach better addresses potential confounding and dropout patterns, providing adjusted estimates of group differences over time (Figures 8A–D, Supplementary Table 4).

**Figure 7:**
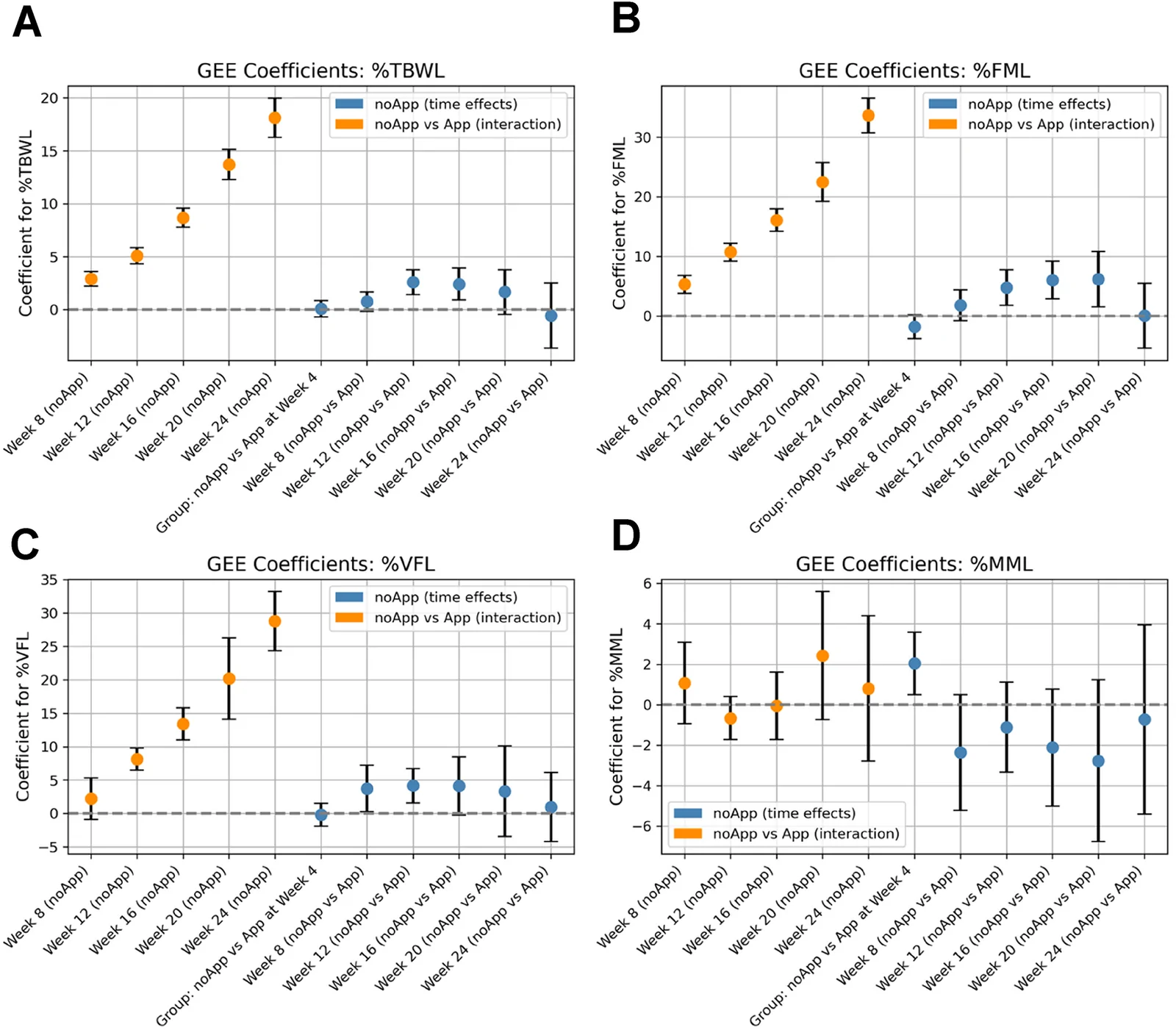
Forest plots of generalized estimating equations (GEE) model coefficients for body composition outcomes. The plots illustrate the longitudinal effects of a lifestyle intervention with the NEP App (App) vs. without the App (noApp) on body composition outcomes over 24 weeks, adjusted for age, BMI, and sex. Blue dots represent time effects within the App group, and orange dots represent group-by-time interaction effects (App vs. no-App), with 95% confidence intervals (CIs). Bars crossing the vertical zero line indicate nonsignificant effects (adjusted *p* > 0.05, Benjamini-Hochberg FDR). **(A)** %TBWL: Significant time effects from week 8 onward (adjusted *p*-values from 1.73 × 10^−15^ to 2.59 × 10^−81^) and group-by-time interactions at weeks 12 (adjusted *p* = 0.00024) and 16 (adjusted *p* = 0.027), with no significant main group effect (adjusted *p* = 1.0). **(B)** %FML: Strong time effects from week 8 (adjusted *p* = 4.75 × 10^−11^ to 1.94 × 10^−113^) and significant group-by-time interactions at weeks 12 (adjusted *p* = 0.028) and 16 (adjusted *p* = 0.0032), with a nonsignificant main group effect (adjusted *p* = 1.0). **(C)** %VFL: Significant time effects from week 12 (adjusted *p*-values from 2.76 × 10^−21^ to 4.33 × 10^−36^) and a group by-time interaction at week 12 (adjusted *p* = 0.024), with a nonsignificant main group effect (adjusted *p* = 1.0). **(D)** %MML: No significant time effects or group-by-time interactions (all adjusted *p* = 1.0), with a nonsignificant main group effect (adjusted *p* = 0.14).

**Figure 8:**
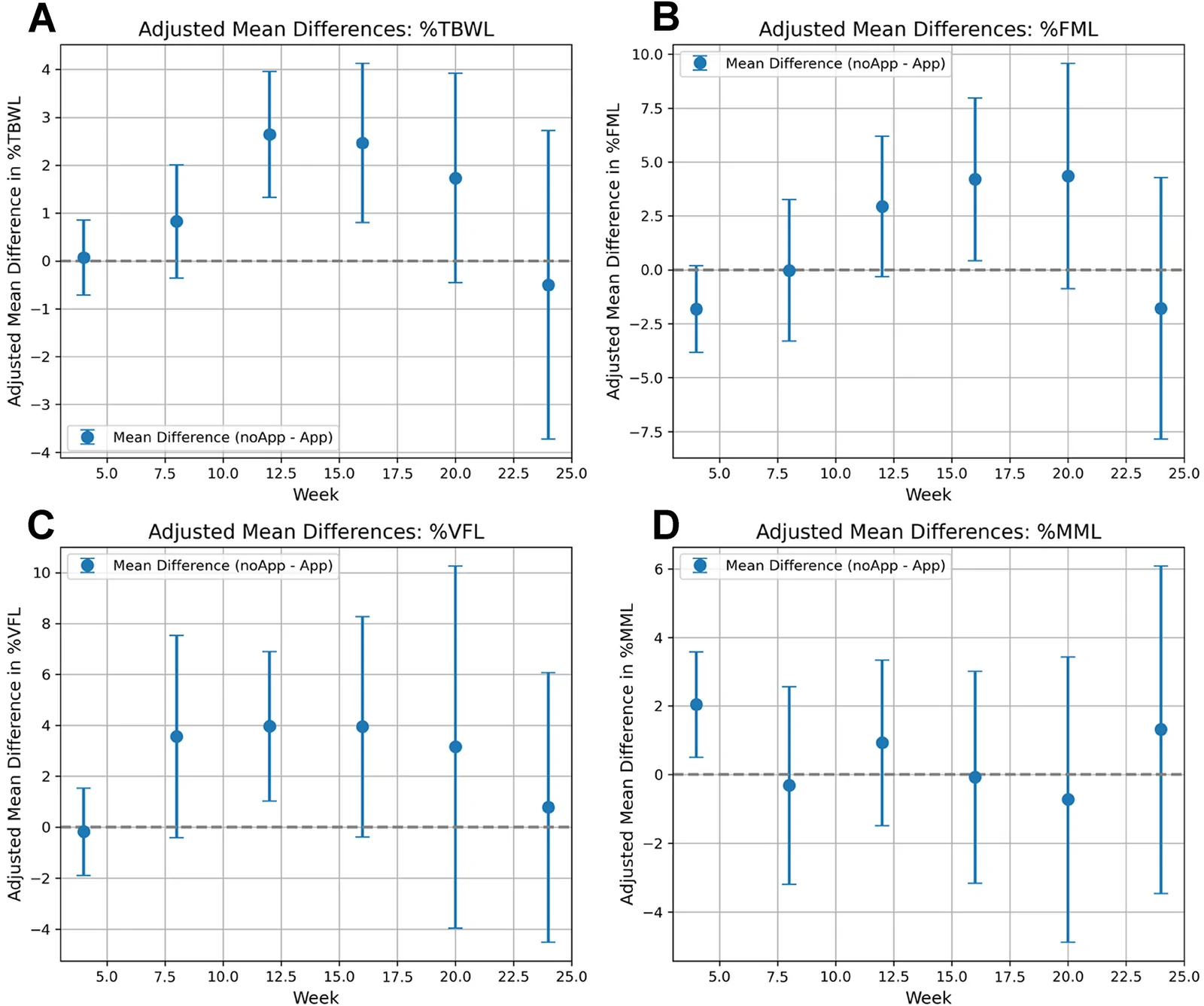
Forest plots of adjusted mean differences for body composition outcomes. The plots illustrate the adjusted mean differences in body composition outcomes between the NEP App (App) and no-App groups over 24 weeks, derived from Generalized Estimating Equations (GEE) models adjusted for age, BMI, and sex, with missing data handled via Multiple Imputation by Chained Equations (MICE) and Inverse Probability Weighting (IPW). Each point represents the mean difference (App minus no-App) at weeks 4, 8, 12, 16, 20, and 24, with 95% confidence intervals (CIs). Bars crossing the vertical zero line indicate nonsignificant differences (adjusted *p* > 0.05, Benjamini-Hochberg FDR). **(A)** %TBWL: Significant mean differences at weeks 12 (adjusted *p* = 0.00024) and 16 (adjusted *p* = 0.027), with nonsignificant differences at weeks 4, 8, 20, and 24 (adjusted *p* ≥ 1.0). **(B)** %FML: Significant mean differences at weeks 12 (adjusted *p* = 0.028) and 16 (adjusted *p* = 0.0032), with nonsignificant differences at weeks 4, 8, 20, and 24 (adjusted *p* ≥ 0.14). **(C)** %VFL: Significant mean difference at week 12 (adjusted *p* = 0.024), with nonsignificant differences at weeks 4, 8, 16, 20, and 24 (adjusted *p* ≥ 0.55). **(D)** %MML: Nonsignificant mean differences at all time points (adjusted *p* = 1.0).

In the GEE analysis for %TBWL, significant time effects were observed from week 8 onward in the App group (coefficients: 2.905 to 18.123, adjusted *p*-values: 1.73 × 10^−15^ to 2.59 × 10^−81^), reflecting progressive weight loss. The main group effect was nonsignificant (coefficient: 0.067, adjusted *p* = 1.0), with an adjusted mean difference at week 4 of 0.067% (95% CI: −0.717 to 0.852). Significant group-by-time interactions emerged at weeks 12 (coefficient: 2.576, adjusted *p* = 0.00024) and 16 (coefficient: 2.398, adjusted *p* = 0.027), with adjusted mean differences of 2.643% (95% CI: 1.324 to 3.962) and 2.465% (95% CI: 0.803 to 4.127), respectively. These indicate that, after adjustment, the App group showed greater %TBWL at these points compared to the no-App group. No significant interactions were found at weeks 8, 20, or 24 (adjusted *p* ≥ 1.0). Age was marginally associated (coefficient: −0.055, adjusted *p* = 0.627), while BMI and sex were not (adjusted *p* = 1.0).

For %FML, significant time effects occurred from week 8 onward (coefficients: 5.299 to 33.649, adjusted *p*-values: 4.75 × 10^−11^ to 1.94 × 10^−conf^). The main group effect was nonsignificant (coefficient: −1.815, adjusted *p* = 1.0), with a week 4 adjusted mean difference of −1.815% (95% CI: −3.825 to 0.194). Significant interactions were at weeks 12 (coefficient: 4.752, adjusted *p* = 0.028) and 16 (coefficient: 6.014, adjusted *p* = 0.0032), with adjusted mean differences of 2.937% (95% CI: −0.322 to 6.196) and 4.198% (95% CI: 0.422 to 7.974). Interactions at other weeks were nonsignificant (adjusted *p* ≥ 0.141). Covariates were not significant (adjusted *p* = 1.0).

For %VFL, time effects were significant from week 12 (coefficients: 8.121 to 28.779, adjusted *p*-values: 2.76 × 10^−21^ to 4.33 × 10^−36^). The main group effect was nonsignificant (coefficient: −0.179, adjusted *p* = 1.0), with a week 4 adjusted mean difference of −0.179% (95% CI: −1.886 to 1.528). A significant interaction occurred at week 12 (coefficient: 4.139, adjusted *p* = 0.024), with an adjusted mean difference of 3.960% (95% CI: 1.024 to 6.896). Other interactions were nonsignificant (adjusted *p* ≥ 0.550). BMI was significantly associated (coefficient: −0.443, adjusted *p* = 0.00036), but age and sex were not (adjusted *p* = 1.0).

For %MML, no significant time effects or interactions were observed (coefficients: −0.671 to 2.424, adjusted *p* = 1.0; main group effect coefficient: 2.041, adjusted *p* = 0.140; week 4 adjusted mean difference: 2.041%, 95% CI: 0.502 to 3.580). Adjusted mean differences ranged from −0.728% to 1.311% across time points. BMI was associated (coefficient: −0.477, adjusted *p* = 0.0015), but age and sex were not (adjusted *p* = 1.0).

The differences between unadjusted and GEE results likely stem from adjustments for baseline imbalances (e.g., age) and handling of missing data, which may have influenced the no-App group's apparent advantage in raw means.

### Exploratory equity analysis

Only two participants in the App group used the Spanish-language version of the RightBMI app; their individual weight-loss trajectories were consistent with the overall App group. Formal statistical comparison by preferred language was therefore not performed. As a related exploratory analysis relevant to health equity, we compared outcomes between self-identified Hispanic vs. non-Hispanic participants within the App group (*n* = 63 per group). Baseline characteristics were well balanced. No statistically significant differences in %TBWL were observed at any time point. A statistically significant difference in %FML was observed at week 12 favoring non-Hispanic users (*p* = 0.0496), though this was not sustained at later time points. Hispanic participants demonstrated significantly lower engagement (median 1.0 vs. 2.0 weeks with available data, *p* = 0.0092). Full results are provided in Supplementary Table 5.

### Patient experience and usability

The App group reported high user satisfaction (mean score: 8.76 ± 1.1), with a high average session duration (7.19 min). User engagement metrics showed a 90.9% return rate and a low bounce rate of 9.6%, indicating strong adherence and usability.

## Discussion

This prospective study evaluated the RightBMI App, an autonomous, protein-focused lifestyle intervention, vs. provider-delivered plans in MBS candidates. A key objective was to assess progression to MBS clearance. By week 24, the App group achieved a 90% clearance rate (72/80) vs. 71.25% (57/80) in the provider-led group (*p* = 0.0023, odds ratio = 3.62, 95% CI: 1.56-Inf), with earlier progress by week 20 (73.75% vs. 66.25%). Low dropout rates (3.75% App, 1.25% no-App) compared to 20%–40% in similar studies (7–10, 14–16, 25, 26) highlight the App's high engagement.

Unadjusted analyses showed greater weight loss in the no-App group at mid-study, but adjusted GEE models showed greater %TBWL and %FML in the App group at weeks 12 and 16. By week 24, point estimates for %TBWL, %FML, %VFL, and %MML were comparable between groups. However, these cross-sectional comparisons at the final time point are limited by substantial attrition, with only 8 patients remaining in the App group and 22 in the no-App group. When analyzed using time-to-event methods that account for informative censoring due to MBS clearance, the App group demonstrated comparable overall effectiveness to provider-delivered care. This supports the robustness of the observed convergence in outcomes by week 24 and provides a more reliable assessment of treatment effect than cross-sectional comparisons at the final time point alone. Notably, at week 8, %FML and %VFL (but not %MML) were linearly associated with %TBWL, suggesting that weight loss primarily involved fat mass.

### Efficacy and comparison to other interventions

The RightBMI App group achieved a mean %TBWL of 19.9% (24.7 kg, 4.89 kg/m^2^ BMI reduction, 35.8% fat loss) in 24 weeks, outperforming other mobile health interventions, which typically yield 0.5–5.5 kg weight loss, 0.4–1.5 kg/m^2^ BMI reduction, and 0.46%–0.79% fat loss (7–10, 14–16, 25, 26). These mid-study differences likely stem from its autonomous, protein-focused design. Unlike pharmacological interventions such as dual GLP-1/GIP agonists (e.g., Tirzepatide) (27), patients using the App achieved comparable weight loss in a shorter timeframe while preserving lean mass (17, 28–30).

The App's autonomy minimizes provider bias and addresses gaps in obesity care (31). Its bilingual (English/Spanish) interface enhances accessibility for diverse populations (32, 33). Exploratory analyses suggested largely equitable weight loss outcomes between Hispanic and non-Hispanic App users, although Hispanic participants showed lower engagement.

The App's standardized delivery aligns with evidence-based weight loss strategies (34). This protein-centric approach, which calculates an individualized daily protein target (approximately 1 g per kg of ideal body weight, adjusted based on prior bariatric research) and dynamically distributes it across the patient's reported sleep/wake cycle and eating patterns, likely contributed to the App group's greater early %TBWL and %FML, high engagement, and effective muscle preservation (17–19).

The user-centered design, which visualizes portion sizes in forkfuls and allows for flexible meal planning, contributed to high adherence and user satisfaction (8.76/10) (35–37).

### Visual portion guidance (“Forkfuls”)

A distinctive feature of the RightBMI platform is the use of visual portion sizes expressed in “forkfuls” rather than rigid calorie counting or food weighing. This approach was developed from extensive clinical experience in bariatric care and translated into the digital algorithm. By converting calculated protein targets into simple, visual, and easily reproducible units, the application aims to reduce cognitive burden while maintaining accuracy and consistency. Patients receive individualized portion guidance that adjusts automatically based on their protein goal, chosen products, and changes in weight or behavior. While visual portion estimation methods have been used in clinical practice, the integration of this approach into a fully autonomous, protein-centric digital platform with dynamic daily adjustment represents a practical innovation.

The app's focus on structured protein intake rather than calorie counting reduces the cognitive and logistical burdens of weight management (38, 39). The App's standardized, bias-free approach contrasts with traditional programs that can vary based on provider expertise (38, 39). Our results represent a significant advancement over other mobile interventions and align with calls for multidisciplinary, individualized approaches to obesity care (38, 40).

Adherence was assessed through a combination of application engagement metrics and body composition-derived indicators, supplemented by clinical follow-up for patients within the bariatric practice. This approach is consistent with established frameworks for evaluating digital behavior change interventions (23) and with recent scoping reviews examining adherence measurement in mHealth physical activity studies (24). While these methods provide practical signals of compliance, they do not constitute direct verification of dietary or exercise behavior.

### Addressing potential socioeconomic barriers

We recognize the potential for socioeconomic selection bias given that the study involved use of a body composition scale and exercise recommendations. To mitigate financial barriers, participants who could not afford one of the recommended body composition scales were provided one free of charge by the study. Additionally, the app's exercise module included aerobic activity options that did not require specialized gym equipment. Clinical staff also provided in-person education on scale use and portion measurement. While complete digital health equity remains challenging, these measures were applied uniformly across both study groups. As noted in prior work, well-intentioned digital health interventions can inadvertently widen disparities if equity considerations are not explicitly addressed during design and implementation (41).

### Study limitations

This study has several limitations. First, findings may have limited generalizability, as the study was conducted at a single center among patients willing to use a smartphone app and protein supplements. Second, this was a prospective quasi-experimental study with sequential group allocation rather than randomization; although statistical adjustments were applied, residual confounding cannot be excluded. Third, the high rate of progression to MBS after Week 8 created informative censoring and reduced sample sizes at later time points. To mitigate the impact of this attrition, we performed a time-to-event analysis (Kaplan–Meier and Cox proportional hazards models) as the primary sensitivity analysis. Fourth, only two participants used the Spanish-language version of the app, limiting conclusions regarding language-based equity. Fifth, adherence was assessed through application engagement metrics and body composition trends rather than direct objective measures such as food diaries or wearable devices. Inferring behavioral compliance solely from weight-loss or fat-loss outcomes introduces a degree of circularity; the indicators used in this study are indirect and do not constitute objective verification of protein intake or exercise adherence. Additionally, body composition was measured using consumer-grade bioimpedance scales. Although participants followed a standardized home measurement protocol and each used the same scale model throughout the study (78.2% used the Renpho scale), these devices have known limitations in absolute precision compared with laboratory methods (36–38). Intra-individual longitudinal trends were therefore prioritized over absolute values.

### Conclusion and future directions

The RightBMI App is a clinically effective, autonomous lifestyle intervention that produced greater mid-study weight and fat loss after adjustment and performed comparably to provider-led counseling on the primary clinical endpoint of time to ≥10% TBWL. Both groups achieved comparable, significant weight loss by week 24, with the App successfully preserving muscle mass, a critical factor for long-term metabolic health (10, 27–29). With a cost-effective and scalable approach, the App matches the efficacy of pharmacological interventions in a shorter time frame, while high user satisfaction and engagement underscore its usability (16, 34–36). By standardizing care and enhancing accessibility, the App effectively mitigates obesity stigma and healthcare disparities (25, 31, 32).

Future research should conduct large-scale, multicenter Randomized Controlled Trials (RCTs) with extended follow-up to validate the long-term durability of the App's benefits. Integrating behavioral reward systems could further enhance adherence (34–36). We also recommend exploring the synergistic effects of the App with GLP-1/GIP receptor agonists to optimize weight management by combining enhanced fat loss with muscle preservation (10, 16, 27–29). This would expand accessibility and provide a comprehensive approach to obesity care.

## Data Availability

The raw data supporting the conclusions of this article will be made available by the authors, without undue reservation.
