# Supplementary material for: Autonomous digital lifestyle intervention for obesity: a comparative study of software-generated vs. provider-delivered body composition outcomes
Source: Front Digit Health. 2026 Oct 23;8:1874279. doi: 10.3389/fdgth.2026.1874279 (PMC13741800; doi:10.3389/fdgth.2026.1874279)
Supplement: Supplementary file 1 [file Supplementaryfile1.docx]

**A**

**B**

**C**

**D**

**Supplemental Figure 1: Principal component analysis (PCA) with explained variance for Week 8 %TBWL, %FML, %VFL and %MML with measurement variables: type of Intervention (App or no-App), Age, BMI and Sex.** **A:** PCA with explained variance for Week 8 %TBWL. 79.4% of the variance was explained by type of Intervention and 20.4% by age. **B:** PCA with explained variance for Week 8 %FML. 78.2% of variance was explained by type of Intervention and 21.6% by age. **C:** PCA with explained variance for Week 8 %VFL. 78% of variance was explained by type of Intervention and 21.9% by age. **D:** PCA with explained variance for Week 8 %MML. 78.1% of variance was explained by type of Intervention and 21.7% by age. Sex accounts for 0% and BMI for 0.1% of each explained variance.

70

60

30

15

Sample Size

**Supplemental Figure 2: Weight Loss Parameter for all Week timepoints for the complete App group vs. the complete no App group.** The size of each bubble shows the patient sample size used for each % Loss calculation. At week 24 sample sizes for both groups become statistically insignificant because of the high number of program dropouts. For both groups % FML and % VFL was significantly greater (1.5 to 2-fold) than %TBWL. % MML was the lowest compared to all other 3 parameters and did not have a positive linear correlation with follow-up as the other 3 parameters had.

**A**

**B**

**C**

**D**

**Supplemental Figure 3: Weight Loss Parameter for all timepoints between weeks 4 and 24 for the noApp group Surgeon supervised cohort (orange) vs the Physician Assistant (PA) supervised cohort for A: %TBWL, B: %FML, C: %VFL, D: %MML.** None of the weight loss parameters, %TBWL, %FML, %MML or %VFL showed any statistically significant difference between the two groups at any timepoint.
