# Supplementary material for: Autonomous digital lifestyle intervention for obesity: a comparative study of software-generated vs. provider-delivered body composition outcomes
Source: Front Digit Health. 2026 Oct 23;8:1874279. doi: 10.3389/fdgth.2026.1874279 (PMC13741800; doi:10.3389/fdgth.2026.1874279)
Supplement: Supplementary file 2 [file Table1.docx]

**Supplemental Table 1: App vs NoApp- Unadjusted Mean Comparisons of Body composition Outcomes**

| **%TBWL** | **App mean** | **App stdev** | **n** | **Attrition %** | **NoApp mean** | **NoApp stdev** | **n** | **Attrition %** | **p-value** | **Test Used** | **BH Corrected p-value** |
| --- | --- | --- | --- | --- | --- | --- | --- | --- | --- | --- | --- |
| **week4** | 3.40 | 2.17 | 73 | 9 | 3.29 | 3.01 | 71 | 11 | 0.80 | t-test | 0.96 |
| **week8** | 6.24 | 3.99 | 60 | 25 | 7.32 | 4.19 | 60 | 25 | 0.15 | t-test | 0.23 |
| **week12** | 8.50 | 4.18 | 47 | 41 | 11.19 | 5.09 | 40 | 50 | 0.01 | t-test | **0.02** |
| **week16** | 11.61 | 4.44 | 33 | 59 | 15.42 | 6.19 | 34 | 58 | 0.01 | t-test | **0.02** |
| **week20** | 15.46 | 6.60 | 21 | 74 | 19.75 | 6.40 | 27 | 66 | 0.03 | t-test | 0.06 |
| **week24** | 20.01 | 7.54 | 8 | 90 | 19.97 | 9.76 | 23 | 71 | 0.99 | t-test | 0.99 |
|  |  |  |  |  |  |  |  |  |  |  |  |
| **%FML** | **App mean** | **App stdev** | **n** | **Attrition %** | **NoApp mean** | **NoApp stdev** | **n** | **Attrition %** | **p-value** | **Test Used** | **BH Corrected p-value** |
| **week4** | 7.40 | 4.74 | 53 | 34 | 5.08 | 8.62 | 61 | 24 | 0.04 | Mann-Whitney U | 0.12 |
| **week8** | 11.56 | 10.49 | 45 | 44 | 12.70 | 14.15 | 54 | 33 | 0.42 | Mann-Whitney U | 0.51 |
| **week12** | 18.19 | 7.79 | 35 | 56 | 20.89 | 13.76 | 37 | 54 | 0.08 | Mann-Whitney U | 0.13 |
| **week16** | 22.70 | 9.33 | 27 | 66 | 28.76 | 15.07 | 32 | 60 | 0.01 | Mann-Whitney U | 0.03 |
| **week20** | 26.81 | 16.21 | 17 | 79 | 35.47 | 14.62 | 24 | 70 | 0.09 | t-test | 0.13 |
| **week24** | 36.81 | 13.50 | 7 | 91 | 35.74 | 21.72 | 21 | 74 | 0.90 | t-test | 0.90 |
|  |  |  |  |  |  |  |  |  |  |  |  |
| **%VFL** | **App mean** | **App stdev** | **n** | **Attrition %** | **NoApp mean** | **NoApp stdev** | **n** | **Attrition %** | **p-value** | **Test Used** | **BH Corrected p-value** |
| **week4** | 4.96 | 4.63 | 52 | 35 | 5.08 | 5.15 | 33 | 59 | 0.81 | Mann-Whitney U | 0.89 |
| **week8** | 7.50 | 15.42 | 43 | 46 | 11.54 | 8.54 | 45 | 44 | 0.24 | Mann-Whitney U | 0.36 |
| **week12** | 13.72 | 8.49 | 34 | 58 | 17.81 | 8.90 | 32 | 60 | 0.06 | t-test | 0.36 |
| **week16** | 18.63 | 9.54 | 26 | 68 | 22.92 | 14.49 | 26 | 68 | 0.21 | t-test | 0.36 |
| **week20** | 21.95 | 24.07 | 16 | 80 | 29.72 | 12.72 | 23 | 71 | 0.20 | t-test | 0.36 |
| **week24** | 31.99 | 20.40 | 6 | 93 | 32.97 | 12.53 | 19 | 76 | 0.89 | t-test | 0.89 |
|  |  |  |  |  |  |  |  |  |  |  |  |
| **%MML** | **App mean** | **App stdev** | **n** | **Attrition %** | **NoApp mean** | **NoApp stdev** | **n** | **Attrition %** | **p-value** | **Test Used** | **BH Corrected p-value** |
| **week4** | -1.30 | 3.24 | 51 | 36 | 1.10 | 4.63 | 61 | 24 | 0.01 | Mann-Whitney U | 0.05 |
| **week8** | 0.56 | 9.09 | 44 | 45 | -0.87 | 9.36 | 54 | 33 | 0.96 | Mann-Whitney U | 0.96 |
| **week12** | -2.12 | 6.57 | 33 | 59 | -1.66 | 10.98 | 37 | 54 | 0.84 | t-test | 0.96 |
| **week16** | -1.64 | 8.15 | 27 | 66 | -2.48 | 12.53 | 32 | 60 | 0.77 | t-test | 0.96 |
| **week20** | 1.25 | 12.77 | 17 | 79 | -1.97 | 10.98 | 24 | 70 | 0.40 | t-test | 0.96 |
| **week24** | -0.09 | 14.02 | 7 | 91 | 0.70 | 12.33 | 21 | 74 | 0.89 | t-test | 0.96 |

**Supplemental Table 2: Surgeon vs Physician Assistant: Unadjusted Mean Comparisons of Body composition Outcomes**

| **%TBWL** | **Surgeon mean** | **Surgeon stdev** | **n** | **Attrition %** | **PA mean** | **PA stdev** | **n** | **Attrition %** | **p-value** | **Test Used** | **BH Corrected p-value** |
| --- | --- | --- | --- | --- | --- | --- | --- | --- | --- | --- | --- |
| **week4** | 4.32 | 2.62 | 50 | 9 | 3.15 | 2.02 | 21 | 16 | 0.06 | t-test | 0.09 |
| **week8** | 7.98 | 2.89 | 39 | 29 | 5.84 | 4.36 | 21 | 16 | 0.06 | t-test | 0.09 |
| **week12** | 10.28 | 4.07 | 26 | 53 | 8.48 | 4.05 | 14 | 44 | 0.19 | t-test | 0.22 |
| **week16** | 12.43 | 3.24 | 24 | 56 | 11.63 | 4.67 | 10 | 60 | 0.64 | t-test | 0.64 |
| **week20** | 20.29 | 5.32 | 20 | 64 | 14.02 | 6.09 | 7 | 72 | 0.04 | t-test | 0.09 |
| **week24** | 27.06 | 5.45 | 17 | 69 | 15.78 | 5.00 | 6 | 76 | 0.02 | t-test | 0.09 |
|  |  |  |  |  |  |  |  |  |  |  |  |
| **%FML** | **Surgeon mean** | **Surgeon stdev** | **n** | **Attrition %** | **PA mean** | **PA stdev** | **n** | **Attrition %** | **p-value** | **Test Used** | **BH Corrected p-value** |
| **week4** | 8.29 | 5.17 | 40 | 27 | 6.97 | 4.54 | 21 | 16 | 0.35 | t-test | 0.64 |
| **week8** | 13.82 | 8.39 | 35 | 36 | 10.32 | 11.43 | 19 | 24 | 0.43 | Mann-Whitney U | 0.64 |
| **week12** | 18.57 | 7.37 | 22 | 60 | 17.94 | 8.23 | 15 | 40 | 0.82 | t-test | 0.90 |
| **week16** | 22.35 | 6.38 | 20 | 64 | 22.87 | 10.67 | 12 | 52 | 0.90 | t-test | 0.90 |
| **week20** | 35.95 | 6.81 | 15 | 73 | 22.65 | 17.74 | 9 | 64 | 0.13 | t-test | 0.40 |
| **week24** | 46.44 | 3.20 | 15 | 73 | 29.59 | 13.97 | 6 | 76 | 0.10 | t-test | 0.40 |
|  |  |  |  | 100 |  |  |  |  |  |  |  |
| **%VFL** | **Surgeon mean** | **Surgeon stdev** | **n** | **Attrition %** | **PA mean** | **PA stdev** | **n** | **Attrition %** | **p-value** | **Test Used** | **BH Corrected p-value** |
| **week4** | 6.27 | 4.05 | 21 | 62 | 4.36 | 4.81 | 12 | 52 | 0.17 | t-test | 0.31 |
| **week8** | 11.30 | 4.87 | 29 | 47 | 5.46 | 18.58 | 16 | 36 | 0.11 | Mann-Whitney U | 0.31 |
| **week12** | 13.93 | 8.50 | 19 | 65 | 13.57 | 8.71 | 13 | 48 | 0.91 | t-test | 0.91 |
| **week16** | 17.73 | 6.47 | 17 | 69 | 19.10 | 10.98 | 9 | 64 | 0.73 | t-test | 0.88 |
| **week20** | 31.98 | 9.97 | 15 | 73 | 15.93 | 28.35 | 8 | 68 | 0.21 | t-test | 0.31 |
| **week24** | 44.28 | 15.79 | 13 | 76 | 19.70 | 18.37 | 6 | 76 | 0.15 | t-test | 0.31 |
|  |  |  |  |  |  |  |  |  |  |  |  |
| **%MML** | **Surgeon mean** | **Surgeon stdev** | **n** | **Attrition %** | **PA mean** | **PA stdev** | **n** | **Attrition %** | **p-value** | **Test Used** | **BH Corrected p-value** |
| **week4** | -2.35 | 3.83 | 40 | 27 | -0.87 | 2.92 | 21 | 16 | 0.14 | t-test | 0.82 |
| **week8** | 0.71 | 11.98 | 35 | 36 | 0.48 | 7.43 | 19 | 24 | 0.94 | Mann-Whitney U | 0.94 |
| **week12** | -1.93 | 8.55 | 22 | 60 | -2.23 | 5.30 | 15 | 40 | 0.91 | t-test | 0.94 |
| **week16** | -3.50 | 9.97 | 20 | 64 | -0.71 | 7.22 | 12 | 52 | 0.41 | t-test | 0.82 |
| **week20** | -3.36 | 14.22 | 15 | 73 | 3.76 | 11.83 | 9 | 64 | 0.29 | t-test | 0.82 |
| **week24** | -1.38 | 20.25 | 15 | 73 | 0.87 | 10.81 | 6 | 76 | 0.86 | t-test | 0.94 |

**Supplemental Table 3: Cox Proportional Hazards Model for Time to Achievement of ≥10% Total Body Weight Loss**

| **Variable** | **Coefficient** | **Hazard Ratio (95% CI)** | **p-value** |
| --- | --- | --- | --- |
| **App vs NoApp (ref)** | -0.19 | **0.83 (0.51 - 1.33)** | 0.433 |
| Age (per 1-year increase) | -0.001 | 1.00 (0.98 -1.02) | 0.953 |
| BMI (per 1 kg/m² increase) | 0.011 | 1.01 (0.98 -1.04) | 0.492 |
| Gender (Male vs Female) | -0.87 | 0.42 (0.15 -1.16) | 0.093 |

**Note:** Model adjusted for age, BMI, and sex. A minor violation of the proportional hazards assumption was detected for BMI (p = 0.048); however, this did not materially affect the estimate for the primary exposure (App vs NoApp). The proportional hazards assumption held for the App variable (p = 0.21).

**Supplemental Table 4: All GEE models results**

| **Outcome** | **Parameter** | **GEE Coefficient** | **Std Error** | **Z** | **P>\|z\|** | **Bonferroni Adjusted p-value** | **CI Lower** | **CI Upper** | **Week** | **Mean Adjusted Difference** | **CI Lower** | **CI Upper** |
| --- | --- | --- | --- | --- | --- | --- | --- | --- | --- | --- | --- | --- |
| %TBWL | Intercept | 5.733 | 2.680 | 2.139 | 0.032 | 0.486 | 0.480 | 10.986 |  |  |  |  |
| %TBWL | time_cat[T.8] | 2.905 | 0.351 | 8.288 | 1.15E-16 | **1.73E-15** | 2.218 | 3.592 |  |  |  |  |
| %TBWL | time_cat[T.12] | 5.087 | 0.387 | 13.135 | 2.07E-39 | **3.10E-38** | 4.328 | 5.846 |  |  |  |  |
| %TBWL | time_cat[T.16] | 8.666 | 0.459 | 18.862 | 2.35E-79 | **3.53E-78** | 7.765 | 9.566 |  |  |  |  |
| %TBWL | time_cat[T.20] | 13.696 | 0.733 | 18.673 | 8.16E-78 | **1.22E-76** | 12.259 | 15.134 |  |  |  |  |
| %TBWL | time_cat[T.24] | 18.123 | 0.942 | 19.240 | 1.73E-82 | **2.59E-81** | 16.277 | 19.969 |  |  |  |  |
| %TBWL | group | 0.067 | 0.400 | 0.169 | 0.866 | 1 | -0.717 | 0.852 | 4 | 0.067 | -0.717 | 0.852 |
| %TBWL | group:time_cat[T.8] | 0.755 | 0.470 | 1.608 | 0.108 | 1 | -0.165 | 1.675 | 8 | 0.823 | -0.359 | 2.004 |
| %TBWL | group:time_cat[T.12] | 2.576 | 0.597 | 4.316 | 1.59E-05 | **0.0002** | 1.406 | 3.745 | 12 | **2.643** | 1.324 | 3.962 |
| %TBWL | group:time_cat[T.16] | 2.398 | 0.769 | 3.117 | 0.002 | **0.027** | 0.890 | 3.905 | 16 | **2.465** | 0.803 | 4.127 |
| %TBWL | group:time_cat[T.20] | 1.660 | 1.072 | 1.548 | 0.122 | 1.000 | -0.442 | 3.761 | 20 | 1.727 | -0.461 | 3.915 |
| %TBWL | group:time_cat[T.24] | -0.569 | 1.572 | -0.362 | 0.718 | 1.000 | -3.650 | 2.513 | 24 | -0.501 | -3.725 | 2.722 |
| %TBWL | age | -0.055 | 0.027 | -2.036 | 0.042 | 0.627 | -0.107 | -0.002 |  |  |  |  |
| %TBWL | bmi | -0.004 | 0.048 | -0.079 | 0.937 | 1.000 | -0.098 | 0.090 |  |  |  |  |
| %TBWL | sex_num | -0.303 | 1.306 | -0.232 | 0.816 | 1.000 | -2.863 | 2.256 |  |  |  |  |
| %FML | Intercept | 9.730 | 5.957 | 1.633 | 0.102 | 1.000 | -1.946 | 21.406 |  |  |  |  |
| %FML | time_cat[T.8] | 5.299 | 0.760 | 6.970 | 3.17E-12 | **4.75E-11** | 3.809 | 6.789 |  |  |  |  |
| %FML | time_cat[T.12] | 10.694 | 0.769 | 13.902 | 6.13E-44 | **9.19E-43** | 9.186 | 12.202 |  |  |  |  |
| %FML | time_cat[T.16] | 16.077 | 0.965 | 16.662 | 2.49E-62 | **3.74E-61** | 14.186 | 17.968 |  |  |  |  |
| %FML | time_cat[T.20] | 22.474 | 1.674 | 13.423 | 4.45E-41 | **6.68E-40** | 19.192 | 25.755 |  |  |  |  |
| %FML | time_cat[T.24] | 33.649 | 1.479 | 22.755 | 1.29E-114 | **1.94E-113** | 30.751 | 36.548 |  |  |  |  |
| %FML | group | -1.815 | 1.025 | -1.771 | 0.077 | 1.000 | -3.825 | 0.194 | 4 | -1.815 | -3.825 | 0.194 |
| %FML | group:time_cat[T.8] | 1.789 | 1.315 | 1.361 | 0.174 | 1.000 | -0.787 | 4.366 | 8 | -0.026 | -3.301 | 3.249 |
| %FML | group:time_cat[T.12] | 4.752 | 1.530 | 3.107 | 0.002 | **0.028** | 1.754 | 7.750 | 12 | **2.937** | -0.322 | 6.196 |
| %FML | group:time_cat[T.16] | 6.014 | 1.624 | 3.703 | 0.000 | **0.003** | 2.830 | 9.197 | 16 | **4.198** | 0.422 | 7.974 |
| %FML | group:time_cat[T.20] | 6.170 | 2.376 | 2.596 | 0.009 | 0.141 | 1.512 | 10.827 | 20 | 4.354 | -0.864 | 9.572 |
| %FML | group:time_cat[T.24] | 0.026 | 2.777 | 0.009 | 0.992 | 1.000 | -5.416 | 5.468 | 24 | -1.789 | -7.846 | 4.267 |
| %FML | age | -0.088 | 0.060 | -1.460 | 0.144 | 1.000 | -0.206 | 0.030 |  |  |  |  |
| %FML | bmi | 0.020 | 0.107 | 0.185 | 0.853 | 1.000 | -0.190 | 0.230 |  |  |  |  |
| %FML | sex_num | 0.905 | 2.290 | 0.395 | 0.693 | 1.000 | -3.584 | 5.394 |  |  |  |  |
| %VFL | Intercept | 26.664 | 6.064 | 4.397 | 0.000 | **0.000** | 14.778 | 38.550 |  |  |  |  |
| %VFL | time_cat[T.8] | 2.209 | 1.580 | 1.398 | 0.162 | 1.000 | -0.887 | 5.306 |  |  |  |  |
| %VFL | time_cat[T.12] | 8.121 | 0.833 | 9.750 | 0.000 | **2.76E-21** | 6.489 | 9.754 |  |  |  |  |
| %VFL | time_cat[T.16] | 13.387 | 1.234 | 10.846 | 0.000 | **3.12E-26** | 10.968 | 15.806 |  |  |  |  |
| %VFL | time_cat[T.20] | 20.185 | 3.113 | 6.485 | 0.000 | **1.34E-09** | 14.084 | 26.285 |  |  |  |  |
| %VFL | time_cat[T.24] | 28.779 | 2.256 | 12.756 | 0.000 | **4.33E-36** | 24.357 | 33.200 |  |  |  |  |
| %VFL | group | -0.179 | 0.871 | -0.205 | 0.837 | 1.000 | -1.886 | 1.528 | 4 | -0.179 | -1.886 | 1.528 |
| %VFL | group:time_cat[T.8] | 3.730 | 1.786 | 2.089 | 0.037 | 0.550 | 0.231 | 7.230 | 8 | 3.551 | -0.419 | 7.522 |
| %VFL | group:time_cat[T.12] | 4.139 | 1.309 | 3.161 | 0.002 | **0.024** | 1.573 | 6.706 | 12 | **3.960** | 1.024 | 6.896 |
| %VFL | group:time_cat[T.16] | 4.119 | 2.222 | 1.854 | 0.064 | 0.956 | -0.235 | 8.473 | 16 | 3.940 | -0.389 | 8.269 |
| %VFL | group:time_cat[T.20] | 3.331 | 3.460 | 0.963 | 0.336 | 1.000 | -3.450 | 10.113 | 20 | 3.153 | -3.966 | 10.271 |
| %VFL | group:time_cat[T.24] | 0.958 | 2.633 | 0.364 | 0.716 | 1.000 | -4.202 | 6.118 | 24 | 0.779 | -4.511 | 6.069 |
| %VFL | age | -0.048 | 0.061 | -0.787 | 0.431 | 1.000 | -0.168 | 0.072 |  |  |  |  |
| %VFL | bmi | -0.443 | 0.105 | -4.225 | 0.000 | **0.000** | -0.649 | -0.238 |  |  |  |  |
| %VFL | sex_num | -1.329 | 2.273 | -0.585 | 0.559 | 1.000 | -5.784 | 3.125 |  |  |  |  |
| %MML | Intercept | 21.904 | 6.605 | 3.317 | 0.001 | **0.014** | 8.959 | 34.849 |  |  |  |  |
| %MML | time_cat[T.8] | 1.070 | 1.027 | 1.041 | 0.298 | 1.000 | -0.944 | 3.083 |  |  |  |  |
| %MML | time_cat[T.12] | -0.671 | 0.545 | -1.232 | 0.218 | 1.000 | -1.740 | 0.397 |  |  |  |  |
| %MML | time_cat[T.16] | -0.058 | 0.851 | -0.068 | 0.946 | 1.000 | -1.726 | 1.610 |  |  |  |  |
| %MML | time_cat[T.20] | 2.424 | 1.614 | 1.502 | 0.133 | 1.000 | -0.740 | 5.587 |  |  |  |  |
| %MML | time_cat[T.24] | 0.802 | 1.831 | 0.438 | 0.661 | 1.000 | -2.787 | 4.390 |  |  |  |  |
| %MML | group | 2.041 | 0.785 | 2.599 | 0.009 | 0.140 | 0.502 | 3.580 | 4 | 2.041 | 0.502 | 3.580 |
| %MML | group:time_cat[T.8] | -2.362 | 1.461 | -1.616 | 0.106 | 1.000 | -5.226 | 0.502 | 8 | -0.321 | -3.202 | 2.559 |
| %MML | group:time_cat[T.12] | -1.117 | 1.137 | -0.982 | 0.326 | 1.000 | -3.346 | 1.112 | 12 | 0.924 | -1.494 | 3.341 |
| %MML | group:time_cat[T.16] | -2.119 | 1.476 | -1.436 | 0.151 | 1.000 | -5.011 | 0.774 | 16 | -0.078 | -3.163 | 3.008 |
| %MML | group:time_cat[T.20] | -2.769 | 2.039 | -1.358 | 0.174 | 1.000 | -6.765 | 1.226 | 20 | -0.728 | -4.882 | 3.426 |
| %MML | group:time_cat[T.24] | -0.730 | 2.384 | -0.306 | 0.760 | 1.000 | -5.402 | 3.943 | 24 | 1.311 | -3.469 | 6.092 |
| %MML | age | -0.059 | 0.050 | -1.176 | 0.240 | 1.000 | -0.158 | 0.040 |  |  |  |  |
| %MML | bmi | -0.477 | 0.123 | -3.896 | 0.000 | 0.001 | -0.718 | -0.237 |  |  |  |  |
| %MML | sex_num | 2.055 | 1.256 | 1.636 | 0.102 | 1.000 | -0.407 | 4.517 |  |  |  |  |

**Supplemental Table 5: Exploratory Equity Analysis**

| **Variable** | **Hispanic n** | **Hispanic median** | **Hispanic IQR** | **Non Hispanic n** | **Non Hispanic median** | **Non Hispanic IQR** | **Test** | **p-value** | **Effect size Cohens d** |  |  |
| --- | --- | --- | --- | --- | --- | --- | --- | --- | --- | --- | --- |
| Age | 59 | 37.3 | 17.35 | 60 | 37.75 | 14.27 | Mann-Whitney U | 0.7863 | 0.083 |  |  |
| BMI | 60 | 43.75 | 10.75 | 61 | 44 | 8.5 | Mann-Whitney U | 0.7206 | -0.055 |  |  |
| Gender | 63 | 0 | 0 | 63 | 0 | 0 | Mann-Whitney U | 0.0697 | 0.327 |  |  |
|  |  |  |  |  |  |  |  |  |  |  |  |
| **Week** | **Outcome** | **Hispanic n** | **Hispanic Mean** | **Hispanic SD** | **Non Hispanic n** | **Non Hispanic Mean** | **Non Hispanic SD** | **Mean Difference** | **Test** | **p-value** | **Effect size Cohens d** |
| 4 | %TBWL | 25 | 3.32 | 2.39 | 37 | 3.6 | 2.19 | -0.28 | Welch t-test | 0.6464 | -0.122 |
| 4 | %FML | 19 | 7.34 | 5.2 | 34 | 7.43 | 4.55 | -0.09 | Mann-Whitney U | 0.7455 | -0.019 |
| 8 | %TBWL | 22 | 5.94 | 3.92 | 30 | 7.08 | 4.13 | -1.14 | Welch t-test | 0.3141 | -0.283 |
| 8 | %FML | 18 | 8.54 | 14.51 | 27 | 13.58 | 6.16 | -5.05 | Mann-Whitney U | 0.1828 | -0.49 |
| 12 | %TBWL | 14 | 7.35 | 4.05 | 27 | 10 | 3.89 | -2.66 | Mann-Whitney U | 0.0526 | -0.673 |
| 12 | %FML | 12 | 14.5 | 8.05 | 23 | 20.11 | 7.08 | -5.61 | Mann-Whitney U | **0.0496*** | -0.756 |
| 16 | %TBWL | 12 | 10.4 | 4.98 | 20 | 12.72 | 3.67 | -2.32 | Mann-Whitney U | 0.1922 | -0.553 |
| 16 | %FML | 10 | 20.26 | 10.64 | 17 | 24.13 | 8.48 | -3.87 | Mann-Whitney U | 0.4666 | -0.416 |
| 20 | %TBWL | 10 | 13.25 | 5.56 | 9 | 18.16 | 6.68 | -4.91 | Mann-Whitney U | 0.0792 | -0.803 |
| 20 | %FML | 7 | 20.24 | 19.81 | 9 | 31.91 | 11.45 | -11.67 | Mann-Whitney U | 0.1142 | -0.749 |

**Supplemental Table 6: Summary of Features and Weight Loss Outcomes from Recent Reviews on Digital Interventions for Obesity/Weight Management Compared to RightBMI App.**

| **App / Study** | **Human Interaction / Coaching** | **Demographics (Ethnicity/Race/SES)** | **Study Design** | **Control Group** | **Assesses Body Composition (Fat/Muscle)** | **Follow-up Length** | **Nutrition Plan** | **Exercise Plan** | **Mean Weight Loss** | **Mean BMI Reduction** | **Mean % Fat Loss** | **Key Notes** |
| --- | --- | --- | --- | --- | --- | --- | --- | --- | --- | --- | --- | --- |
| AKTIDIET [7-8] | Yes (1 in-person session + motivational messages) | Spain; limited diversity data | RCT | Minimal intervention | No | 6 months | Structured | Structured | ~1–2 kg | 0.76 | N/A | — |
| SoSu-life [7-8] | Minimal (self-guided with feedback) | Denmark; limited diversity data | RCT | No intervention | Yes (body fat %) | 38 weeks | Individualized | Individualized | ~1–2 kg | N/A | 0.46–0.79% | Primarily tracking + feedback |
| My Meal Mate [7-8] | Yes (1 in-person session) | UK; limited diversity data | RCT | Paper diary + in-person | Yes (body fat %) | 6 months | Individualized | Individualized | ~2.2 kg | ~0.8 | 0.46–0.79% | — |
| CogniNU [7] | No | Taiwan; limited diversity data | RCT | No intervention | No | 60 days | None | None | ~1–2 kg | N/A | N/A | Food image recognition |
| CITY [8] | No | US; limited diversity data | RCT | Minimal intervention or coaching | No | 6, 12, 24 months | None | None | ~1.5 kg | N/A | N/A | Self-monitoring focus |
| PRECIOUS [8] | Yes (up to 3 in-person sessions) | Limited data | RCT | Usual care + diary | Yes (weight management) | 3 months | None | None | N/A | N/A | N/A | Biofeedback focus |
| Lose It! [8] | Yes (1-2 in-person sessions) | US; some African American focus | RCT | Another app or waiting list | No | 3, 6 months | None | None | ~1–2 kg | N/A | N/A | — |
| EVIDENT3 [8] | Yes (1 in-person session) | Limited data | RCT | Minimal intervention | No | 3, 12 months | None | None | ~1.8 kg | ~0.7 | N/A | — |
| B-slim [8] | No | Limited data | RCT | Waiting list or coaching | No | 3 months | Structured | Structured | ~0.5 kg | N/A | N/A | — |
| Smart Walk [8] | Yes (1 in-person session) | US; limited diversity data | RCT | Minimal intervention | No | 4, 8 months | None | None | N/A | N/A | N/A | Physical activity focus |
| Top Tips (10TT) [8] | No | Limited data | RCT | Waiting list or another app | No | 3 months | None | None | ~1 kg | N/A | N/A | Dietary intake focus |
| PathMate2 [8] | Yes (2 in-person sessions) | Limited data | RCT | Personal coaching | No | 5.5, 12 months | None | None | N/A | N/A | N/A | Biofeedback |
| WW / Keyto App [8] | Yes (1 in-person session) | US; limited diversity data | RCT | Another app + in-person | No | 3, 6, 12 months | None | None | ~1–2 kg | N/A | N/A | — |
| CALO mama Plus [8] | Yes (1 in-person session) | Limited data | RCT | Waiting list + in-person | No | 3 months | None | None | ~1.5 kg | N/A | N/A | — |
| zanadio [16] | Minimal to moderate (some coaching) | Germany; limited diversity; higher SES bias | RCT | Usual care / minimal | Limited (mainly weight/BMI) | 3–12 months | Structured & personalized | Structured | ~3–6 kg | ~1–2 | N/A | Closest recent competitor |
| FiNC Wellness Coach [8] | No | Limited data | RCT | Waiting list | No | 3 months | None | None | ~1 kg | N/A | N/A | — |
| ImpulsePal [8] | No | Limited data | RCT | Waiting list | No | 3 months | None | None | ~1 kg | 0.4 | N/A | Snack reduction focus |
| W8Loss2Go [8] | Yes (3 in-person sessions) | Limited data | RCT | Personal coaching | No | 6, 12, 18 months | None | None | N/A | N/A | N/A | — |
| TeamBuildr [8] | No | Limited data | RCT | Waiting list | No | 4 months | None | None | ~1–2 kg | N/A | N/A | Physical activity focus |
| Noom Weight [14] | Yes (1:1 coaching + group support) | US; 95% Caucasian; higher education | RCT | Standard care | No | 4 months | Individualized | Individualized | 5.5 kg | 1.5 | N/A | Strong coaching component |
| MyFitnessPal [15] | No (self-guided tracking) | US; mixed race/ethnicity; moderate SES | RCT | Usual care | No | 6 months | Calorie tracking (recent planner feature) | Activity tracking | 1.8 kg | 0.6 | N/A | Primarily self-monitoring tool |
| **RightBMI App (This study)** | **No** – fully autonomous, no provider involvement required* | US (Holyoke, MA); Mean age 41 (SD 12); BMI 43 (SD 7.9); 87.5% female; English/Spanish versions; addresses underserved populations | Prospective quasi-experimental with sequential allocation | Provider-delivered lifestyle intervention | **Yes** (fat mass, visceral fat, muscle mass loss)* | Up to 24 weeks | **Structured, dynamic, protein-focused individualized plans*** | **Structured, individualized*** | **24.7 kg (19% TBWL)*** | **4.89**  **8*** | **35.8%*** | **Fully autonomous + strong muscle preservation**  * |

*Bold type denotes the only app that has (i) nutrition plans that are structured, dynamic, protein focused and individualized, (ii) exercise plans that are structured and individualized, (iii) is fully autonomous with strong muscle preservation focus and (iv) assesses body composition.
